# Renoprotective actions of puerarin and the prospects of kidney-targeted nanodelivery

**DOI:** 10.3389/fphar.2026.1896730

**Published:** 2026-08-10

**Authors:** Yujiao Huang, Jia Zheng, Fengyu Jin, Jun Li, Meifei Lian, Ting Chen, Minghui Jiang, Hongjiao Chen, Yiwen Cao, Atanas G. Atanasov, Zhiguang Qiao, Linyuan Wang

**Affiliations:** 1 School of Chinese Materia Medica, Beijing University of Chinese Medicine, Beijing, China; 2 State Key Laboratory of Macromolecular Drugs and Large-scale Preparation, Liaocheng University, Liaocheng, China; 3 Department of Implant Dentistry, Shanghai Ninth People’s Hospital, Shanghai Jiao Tong University School of Medicine, Shanghai, China; 4 Ludwig Boltzmann Institute Digital Health and Patient Safety, Medical University of Vienna, Spitalgasse, Vienna, Austria; 5 Laboratory of Natural Products and Medicinal Chemistry (LNPMC), Center for Global Health Research, Saveetha Medical College and Hospital, Saveetha Institute of Medical and Technical Sciences (SIMATS), Chennai, India; 6 Institute of Genetics and Animal Biotechnology of the Polish Academy of Sciences, Magdalenka, Poland; 7 Department of Bone and Joint Surgery, Department of Orthopedics, Renji Hospital, Shanghai Jiao Tong University School of Medicine, Shanghai, China; 8 Clinical and Translational Research Center for 3D Printing Technology, Medical 3D Printing Innovation Research Center, Shanghai Ninth People’s Hospital, Shanghai Jiao Tong University School of Medicine, Shanghai, China

**Keywords:** kidney diseases, kidney-targeted, nanodelivery, pharmacological activities, puerarin

## Abstract

Puerarin, a natural isoflavone derived from the roots of kudzu vine, has shown promising protective effects against kidney diseases. It exerts antioxidative, anti-inflammatory, antifibrotic, and antiapoptotic effects that collectively reduce renal injury and slow disease progression. Despite these pharmacological benefits, the therapeutic potential of puerarin is limited by extremely poor solubility, low oral bioavailability, and rapid metabolism. Advances in nanodelivery have provided innovative strategies to address these barriers, including nanoparticles, hydrogels, liposomes, micelles, and dendrimers. Although the applications of puerarin nanodelivery in kidney diseases remain limited, evidence from other pathological models provides valuable guidance for designing kidney-specific approaches. In this review, existing studies on the therapeutic effects of puerarin in renal disorders are integrated with recent progress in puerarin nanodelivery technologies, and the prospects of puerarin-based nanodelivery systems for kidney diseases are discussed.

## 1 Introduction

Puerarin, a natural isoflavone derived from the roots of *Pueraria lobata* and *Pueraria thomsonii* (Yue et al., 2024; Wu et al., 2023), is used in the management of cardiovascular, metabolic, ophthalmological, and neurological disorders (Jiang et al., 2022; Jing X. et al., 2022; Meng et al., 2022; Liu X. et al., 2023). Emerging evidence supports the renoprotective properties of puerarin (Wang D. et al., 2022; Gao et al., 2023; Song et al., 2024). To date, mainstream pharmacotherapies for treating renal diseases, including renin–angiotensin–aldosterone system inhibitors (RAASis), glucocorticoids, and immunosuppressants, are limited by adverse effects and contraindications (Group et al., 2024). RAASis may relieve symptoms but not prevent progression to end-stage renal disease (ESRD) (Ruiz-Ortega et al., 2020). Although glucocorticoids and immunosuppressants reduce proteinuria, they frequently cause adverse reactions, require careful patient selection, and increase healthcare costs (Wang J. Y. et al., 2021). In contrast, many natural products derived from traditional Chinese medicine are characterized by multitarget activities and low toxicity. These properties render them promising alternative therapeutic strategies for symptom management, survival improvement, and the reduction of adverse events while also offering valuable leads for novel drug discovery (Jiang et al., 2024; Zhong et al., 2015; Wang Y. et al., 2022; Tang et al., 2021; Atanasov et al., 2021).

Kidney diseases encompass a broad spectrum of conditions characterized by structural and functional kidney impairment, ranging from acute kidney injury (AKI) to chronic kidney disease (CKD) and from primary nephropathy to secondary nephropathy (Ostermann et al., 2025; Romagnani et al., 2025; Lerner et al., 2021; Hu et al., 2025; Tomita et al., 2020). Despite their heterogeneity, these diseases share common pathological mechanisms, including oxidative stress, inflammation, fibrosis, apoptosis, and lipid metabolism dysregulation, all of which contribute to renal dysfunction (Liu B. et al., 2018; Cai et al., 2024; Chen et al., 2023; Zhou X. et al., 2024; Wang X. W. et al., 2021; Cao et al., 2024; Liu et al., 2022).

The clinical translation of puerarin is limited by poor aqueous solubility, low lipophilicity, and inadequate oral bioavailability (Jing X. et al., 2022). To overcome these barriers, nanodelivery systems, including nanoparticles, hydrogels, liposomes, micelles, and dendrimers, have been developed to increase puerarin solubility and promote targeted accumulation (Jing X. et al., 2022; Zhang, 2019). These strategies not only improve systemic bioavailability but also facilitate site-specific drug delivery (Singh et al., 2013; Chen H. R. et al., 2024), thereby offering important guidance for the renal-targeted transport of puerarin.

Several reviews have summarized the pharmacological effects of puerarin or the application of nanodelivery systems in individual diseases. However, a comprehensive review of the renoprotective effects of puerarin across different kidney diseases and their underlying molecular mechanisms remains lacking. In addition, kidney-targeted delivery strategies for puerarin have received limited attention, and most nanomaterial-based studies have been conducted in non-renal diseases. Therefore, this review summarizes the molecular mechanisms underlying the renoprotective effects of puerarin and provides a prospective overview of kidney-targeting principles and nanomaterial-based strategies for future kidney-targeted puerarin delivery. By integrating pharmacological evidence with emerging nanodelivery concepts, this review aims to provide a framework for the development of kidney-targeted puerarin formulations.

A literature search was conducted in PubMed and Web of Science up to December 2025 using the keywords “puerarin” and “kidney” or “renal”, as well as “puerarin” and nanodelivery-related terms. Original research articles published that focused on the renoprotective effects, mechanisms, or delivery strategies of puerarin were included. Publications unrelated to kidney diseases or puerarin-based nanodelivery, conference abstracts, editorials, and duplicate records were excluded. Retrieved records were assessed for relevance to the scope of this review, and a total of 124 publications were ultimately included.

## 2 Molecular mechanism of puerarin in kidney diseases

The development and progression of kidney diseases involve a complex interplay of diverse pathophysiological processes. Puerarin has multifaceted molecular effects on renal disorders (Table 1). In this section, the therapeutic effects of puerarin, including its antioxidative, anti-inflammatory, and antifibrotic effects and its ability to inhibit apoptosis, regulate autophagy and pyroptosis, modulate lipid metabolism, and enhance renal clearance function, are summarized.

**TABLE 1:** Therapeutic effect of puerarin in kidney disease.

Number	Experimental model	Puerarin dose or formulation, routes	Major effector cells	Effect	Mechanism	*In vivo* or *in vitro*	References
1	MTX transport/nephrotoxicity model; HEK293 and MDCK-MDR1 cells	50 mg/kg i.g. or i.v.; 0.01–100 μM	TECs	Reduced renal MTX accumulation and lowered Scr and BUN; altered MTX pharmacokinetics and renal clearance	Inhibited OAT1/OAT3-mediated renal uptake while reducing nephrotoxicity	*in vivo* and *in vitro*	Liu et al. (2014)
2	MTX-induced renal damage; NRK-52E cells	50 mg/kg i.p.; 25 μM	TECs	Reduced Scr and BUN, promoted MTX elimination	Upregulated renal OAT1/OAT3; BCL6 activation suppressed NF-κB/PGE2 signaling	*in vivo* and *in vitro*	Liu et al. (2018b)
3	Lead-induced nephrotoxicity	100, 200, 300, or 400 mg/kg i.g	-	Dose-dependently improved serum urea, uric acid and creatinine, ROS, GSH and apoptosis	Improved antioxidant activity; activated PI3K/AKT/eNOS/NO; restored Bcl-2/Bax balance and inhibited Cyt c release and caspase-3	*in vivo*	Liu et al. (2012)
4	Renal I/R injury; HK-2 hypoxia/reoxygenation model	50 or 100 mg/kg i.p.; 1 or 10 μM	TECs	Improved Scr and BUN, reduced tubular injury and apoptosis	Activated Nrf2/HO-1 and reduced ER stress proteins GRP78, p-eIF2α, CHOP and caspase-3	*in vivo* and *in vitro*	Wang et al. (2025a)
5	CaOx kidney stone model; COM-treated HK-2 cells injury	10, 50, or 100 mg/kg i.g.; 8, 16, or 32 μM	TECs	Reduced Scr and BUN, CaOx crystal deposition, renal damage, apoptosis, oxidative stress and inflammation	Activated PI3K/AKT signaling; lowered inflammatory/apoptotic responses	*in vivo* and *in vitro*	Xu et al. (2024)
6	db/db mice; high glucose-induced podocyte injury	20 mg/kg i.g 10 μM	Podocytes	Reduced albuminuria and podocyte apoptosis; improved glomerular injury	Directly interacted with Gnai1 and activated cAMP/PKA/CREB signaling	*in vivo* and *in vitro*	Zhu et al. (2022)
7	Cadmium-induced TEC apoptosis	100 μM	TECs	Protected TECs from cadmium cytotoxicity and apoptosis	Restored mitochondrial membrane potential; regulated ANT1, ANT2 and mPTP; reduced Cyt c release, caspase-3 and PARP cleavage; increased Bcl-2/Bax ratio	*in vitro*	Song et al. (2016)
8	STZ-induced DN; high glucose-induced HBZY-1 cells injury	50 or 100 mg/kg; 0–200 μM	MCs	Reduced blood glucose and urinary microalbumin; restored CAT, SOD and GSH; improved renal histology; protected high glucose injured cells	Inhibited p38 MAPK-related inflammatory and fibrotic signaling including TNF-α, CREB/Fos/Jun/MMP9 axis	*in vivo* and *in vitro*	Zhang et al. (2020)
9	STZ-induced DN	140, or 200 mg/kg i.g	-	Reduced blood glucose, triglycerides, total Cholesterol, MDA, BUN, Scr, urinary protein and glomerular ECM; increased SOD, CAT, GSH-Px and NO	Suppressed oxidative stress and downregulated TGF-β1, Smad2, CTGF and FN	*in vivo*	She et al. (2014)
10	High glucose-induced SV40-MES-13 renal MCs	2.5, 5, or 10 μM	MCs	Reduced ROS and MDA and restored SOD and GSH-Px in MCs	Suppressed AGEs/RAGE/PKC β/NOX4 axis	*in vitro*	Wang and Li (2024)
11	Cisplatin-induced nephrotoxicity; HK-2 cells exposed to cisplatin, aristolochic acid I, or citrinin	As a natural hydroxyl radical-scavenging antioxidant	TECs	Puerarin reduced hydroxyl radical-related renal cell injury signals in the screening platform	Activated SIRT1/Nrf2/KEAP1 antioxidant signaling	*in vitro*	Gao et al. (2023)
12	high-fat and high-sugar diet + STZ-induced DN	40 or 80 mg/kg i.g	Podocytes	Attenuated podocyte damage and renal pathological changes	Activated AMPK/Nrf2 pathway and antioxidant defense	*in vivo*	Xue et al. (2025a)
13	STZ-induced DN	20, 40, or 80 mg/kg i.g	-	Improved blood glucose, BUN, Scr, urinary albumin and mitochondrial ultrastructure	Activated SIRT1/FOXO1/PGC-1α and enhanced antioxidant defense	*in vivo*	Xu et al. (2016)
14	STZ-induced DN	100 mg/kg i.p	TECs	Improved body weight, kidney weight, urinary protein, Scr, BUN and renal pathology	Attenuated abnormal eNOS expression	*in vivo*	Zhang et al. (2015)
15	Lead-induced nephrotoxicity	400 mg/kg i.g	-	Reduced renal dysfunction and oxidative injury	Antioxidant and chelating-related protection; lowered ROS, lipid peroxidation and restored antioxidant enzymes	*in vivo*	Wang et al. (2013)
16	STZ-induced DN; high glucose-induced podocytes injury	20 mg/kg i.g.; 1, 10 μM	Podocytes	Reduced albuminuria, superoxide production, glomerular injury and podocyte damage	Activated SIRT1, suppressed NF-κB acetylation and NOX4 expression in podocytes	*in vivo* and *in vitro*	Li et al. (2017)
17	Cadmium chloride-induced nephrotoxicity	100 mg/kg i.g	-	Reduced BUN and renal oxidative stress, improved antioxidant enzyme activities	Antioxidant protection: decreased MDA and restored SOD, CAT and GSH-Px	*in vivo*	Wang et al. (2019a)
18	Cisplatin-induced nephrotoxicity	10, 30, or 50 mg/kg i.v	TECs	Dose-dependently lowered BUN and Scr, reduced inflammatory cytokines	Inhibited TLR4/NF-κB p65 signaling; restored antioxidant systems and reduced MDA and xanthine oxidase	*in vivo*	Ma et al. (2017)
19	LPS-induced Madin-Darby bovine kidney cells injury	50, 100, or 150 μM	Kidney epithelial cells	Improved LDH release and reduced IL-1β, IL-8 and TNF-α	Suppressed TLR4 and phosphorylated NF-κB; NF-κB activator reversed protection	*in vitro*	Zhou et al. (2024b)
20	STZ-induced DN; high glucose-injury HK-2 cells injury	20, 40, or 80 mg/kg i.g 80 mg/L	TECs	Improved renal pathology, apoptosis, blood glucose, Urine Albumin-to-Creatinine Ratio, inflammatory cytokines and body weight	Upregulated miRNA-140/145-5p and reduced TLR4/MyD88/NF-κB p65 activation	*in vivo* and *in vitro*	Xu et al. (2020a)
21	UUO-induced renal inflammation and fibrosis model	50 or 100 mg/kg i.g	TECs	Reduced Scr, BUN, 24 h urinary protein, tubular and glomerular deformation, ECM accumulation and fibrosis	Inhibited NF-κB p65/STAT3 inflammatory signaling and TGF-β1/Smad3; upregulated Smad7	*in vivo*	Wang et al. (2021a)
22	LPS-induced and UUO-induced renal inflammatory model; LPS-treated Raw264.7 macrophages	50 or 100 mg/kg i.p.; 50 μM	Macrophages	Reduced renal inflammation and macrophage M1 polarization	Directly antagonized TLR4/MyD88; inhibited NF-κB p65 and JNK/FOXO1 activation	*in vivo* and *in vitro*	Hu et al. (2024)
23	db/db mice; high glucose-induced podocytes injury	Puerarin 10 mg/kg + arctigenin 20 mg/kg i.g; 10 μM	Podocytes	Combination reduced albuminuria and proteinuria and inflammatory gene expression	Puerarin reduced NF-κB p65 acetylation via SIRT1; arctigenin reduced p65 phosphorylation via PP2A	*in vivo* and *in vitro*	Li et al. (2024)
24	CRF model; LPS/H_2_O_2_-induced HK-2 cells injury	400 mg/kg i.g 50 or 100 μM	TECs	Improved kidney injury and fibrosis markers; inhibited HK-2 apoptosis and pyroptosis	Downregulated lncRNA NEAT1 and inhibited caspase-1/GSDMD-N, IL-1β and IL-18 release	*in vivo* and *in vitro*	Yang et al. (2024a)
25	Renal I/R injury	50 mg/kg i.p	TECs	Reduced Scr, BUN and inflammatory/pyroptotic damage	Suppressed NLRP3/caspase-1/GSDMD pathway	*in vivo*	Wang et al. (2023)
26	Adenine-induced CRF; LPS/H2O2-induced HK-2 cell injury	400 mg/kg i.g.; 100 μM	TECs	Improved renal morphology, fibrosis and reduced TEC pyroptosis	Upregulated miR-342-3p and inhibited TGF-β/Smad axis	*in vivo* and *in vitro*	Yang et al. (2023)
27	STZ-induced DN; high glucose-treated MPC5 podocytes injury	20, 40, or 80 mg/kg; 0.25, 0.5, or 1 mM	Podocytes	Improved podocyte injury, urine protein and inflammatory factor release	Inhibited caspase-1-mediated pyroptosis and reduced GSDMD, IL-1β and IL-18	*in vivo* and *in vitro*	Chen et al. (2024b)
28	STZ-induced DN mice; high glucose-induced MPC-5 podocytes	80 mg/kg; 0.8 mM	Podocytes	Reduced podocyte pyroptosis and restored nephrin, podocin and podocalyxin	Modulated SIRT1/NLRP3/caspase-1 pathway and reduced inflammasome/GSDMD activity	*in vivo* and *in vitro*	Wang et al. (2025b)
29	Renal I/R injury; HK-2 hypoxia/reoxygenation model	50 or 100 mg/kg i.p.; 1 or 10 μM	TECs	Reduced COL deposition, fibrosis, oxidative stress and ferroptosis	Inhibited TLR4/NOX4 pathway and ferroptosis-related injury	*in vivo* and *in vitro*	Jian et al. (2023)
30	UUO-induced renal fibrosis; H2O2-induced TEC injury	50 or 100 mg/kg i.g.; 50 or 100 μM	TECs	Attenuated tubular injury, ECM deposition, fibrosis and TEC apoptosis	Reduced oxidative stress-induced apoptosis via MAPK signaling pathways	*in vivo* and *in vitro*	Zhou et al. (2017)
31	STZ-induced DN injury	100 mg/kg i.p	Podocytes	Attenuated kidney hypertrophy, mesangial expansion, podocyte foot process effacement and albuminuria	Downregulated MMP9 and protected podocyte/glomerular matrix structure	*in vivo*	Zhong et al. (2014)
32	High-fat and high-sucrose diet + STZ-induced DN and high glucose-induced HBZY-1 MCs	100 mg/kg i.g.; 1 or 10 μM	MCs	Improved renal filtration/function and reduced excessive ECM/COL accumulation	Inhibited ferroptosis and profibrotic markers COL I, COL IV, FN,α-SMA and TGF-β	*in vivo* and *in vitro*	Hou et al. (2024)
33	High glucose-induced rat MCs	10, 25, 50, or 100 μM	MCs	Improved MCs proliferation and viability, protected cytoskeleton	Suppressed TGF-β1 overexpression and Smad2/3 nuclear translocation; reduced FN/COL signaling	*in vitro*	Barro et al. (2021)
34	High-fat diet + STZ-induced DN	Canagliflozin 1.5 mg/kg + puerarin 4, 10, 40, or 100 mg/kg i.g	TECs	Alleviated renal lipotoxicity, inflammation and oxidative stress; improved kidney metabolic parameters	Reduced CD36/adipophilin-mediated lipid uptake; activated PPARα and FA oxidation	*in vivo*	Zhu et al. (2023)
35	CaOx crystal-induced renal injury; ethylene glycol oxalate-induced HK-2 cells injury	50 or 100 mg/kg i.g.; 32 μM	TECs	Reduced CaOx-induced TEC autophagy, cytotoxicity, ROS and renal injury	Activated SIRT1-mediated signaling; modulated AKT and p38 MAPK to prevent excessive autophagy	*in vivo* and *in vitro*	Jing et al. (2022b)
36	STZ-induced DN	40 or 80 mg/kg i.g	Podocytes	Improved renal function, podocyte ultrastructure and nephrin, podocin, and podocalyxin levels	Regulated PERK/eIF2α/ATF4-mediated autophagy; increased Beclin-1/LC3II/Atg5 and decreased p62	*in vivo*	Xu et al. (2020b)
37	Cadmium-induced NRK-52E TEC injury	100 μM	TECs	Reduced cadmium-induced ER stress and restored cell homeostasis	Restored autophagic flux; inhibited Nrf2/KEAP1-mediated excessive autophagy and restored RAB7-related flux	*in vitro*	Liu et al. (2020a)
38	STZ-induced DN	100 mg/kg i.g	Podocytes	Reduced Scr, BUN and lipid abnormalities and protected podocyte and renal structure	Activated AMPK/mTOR-mediated autophagy signaling	*in vivo*	Su et al. (2023)
39	Uox^−/−^ spontaneous hyperuricemia	300 mg/kg i.g	TECs	Reduced serum uric acid and alleviated hyperuricemia-associated renal injury, including tubular swelling, cortical vacuolization, glomerular atrophy and inflammatory infiltration	Promoted uric acid excretion; regulated urate transporter-related injury markers	*in vivo*	Xu et al. (2022)

### 2.1 Antioxidative stress

**FIGURE 1:**
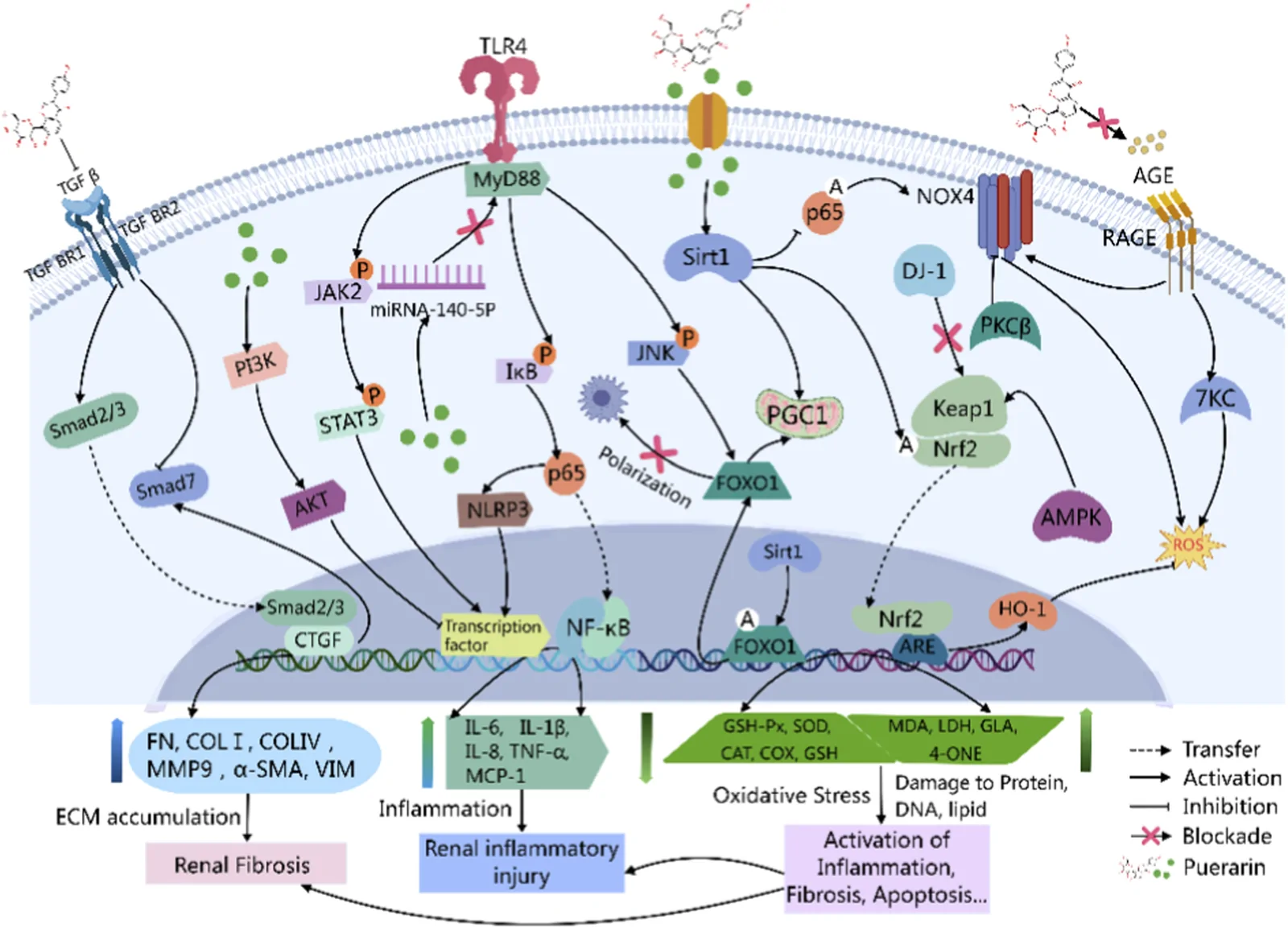
Puerarin attenuates oxidative stress, inflammation, and fibrosis in kidney diseases. 7KC, 7-Ketocholesterol; AGE, Advanced glycation end product; AKT, RAC-alpha serine/threonine-protein kinase; AMPK, 5′-AMP-activated protein kinase; ARE, Antioxidant response element; CAT, Catalase; COL, Collagen; COX, Cyclooxygenase; CTGF, CCN family member 2; DJ-1, Parkinson disease protein 7; FN, Fibronectin; FOXO1, Forkhead box protein O1; GLA, Glycolaldehyde; GSH, Glutathione; GSH-Px, Glutathione peroxidase; HO-1, Heme oxygenase-1; IL, Interleukin; IκB, Inhibitor of NF-κB; JAK2, Tyrosine-protein kinase JAK2; JNK, c-Jun N-terminal kinase; KEAP1, Kelch-like ECH-associated protein 1; LDH, Lactate dehydrogenase; MCP-1, Monocyte chemoattractant protein-1; MDA, Malondialdehyde; MMP9, Matrix metalloproteinase-9; MyD88, Myeloid differentiation primary response protein MyD88; NF-κB, Nuclear factor-κb; NLRP3, LRR and PYD domains-containing protein 3; NOX4, NADPH oxidase 4; Nrf2, Nuclear factor erythroid 2-related factor 2; PGC1, Peroxisome proliferator-activated receptor γ coactivator 1; PI3K, Phosphatidylinositol 3-kinase; PKCβ, Protein kinase C beta type; RAGE, Receptors of advanced glycation end-products; ROS, Reactive oxygen species; SIRT1, Sirtuin-1; Smad, SMAD family member; SOD, Superoxide dismutase; STAT3, Signal transducer and activator of transcription 3; TGF BR, Transforming growth factor beta receptor; TGF β, Transforming growth factor-β; TLR4, Toll-like receptor 4; TNF-α, Tumor necrosis factor-α; VIM, Vimentin; and α-SMA, α-smooth muscles actin.

Oxidative stress plays a pivotal role in both the development and progression of kidney diseases (Duni et al., 2019; Patera et al., 2024; Jha et al., 2018). Targeting oxidative stress is an important therapeutic strategy for the management of renal disorders (Uddin et al., 2021; Verma et al., 2021). Excessive accumulation of reactive oxygen species (ROS) not only directly damages podocytes (Xue S. et al., 2025), mesangial cells (MCs) (Wang and Li, 2024), and renal tubular epithelial cells (TECs) (Gao et al., 2023) but also causes oxidative modifications of proteins, DNA, and lipids, thereby initiating inflammation, apoptosis, and fibrosis and accelerating renal pathology (Cai et al., 2024). Puerarin, a potent isoflavone, exhibits robust antioxidative properties by modulating both enzymatic and nonenzymatic antioxidant defence systems. It increases the activities of superoxide dismutase (SOD), catalase, glutathione peroxidase (GSH-Px), manganese SOD, and lactate dehydrogenase (Xu et al., 2024; She et al., 2014; Xu et al., 2016) and increases glutathione levels. These actions restore oxidative stress markers, such as malondialdehyde (MDA) and 7-ketocholesterol, to baseline levels, reinforcing the antioxidant defence of the kidney (Xu et al., 2024; She et al., 2014; Zhang Z. T. et al., 2021). By scavenging ROS and regulating redox signalling pathways in the kidney (Figure 1), puerarin effectively mitigates oxidative stress and helps preserve renal architecture and glomerular function (Xue S. et al., 2025; Xu et al., 2016; Li et al., 2017).

Sirtuin-1 (SIRT1), an NAD^+^-dependent deacetylase, is broadly involved in cellular oxidative stress, metabolism, and ageing (Almalki and Salman Almujri, 2024; Chen et al., 2025; Guan et al., 2025). Puerarin confers antioxidative protection in various models of renal injury via SIRT1 activation. In streptozotocin (STZ)-induced diabetic nephropathy (DN) in rats, SIRT1 deacetylates and activates Forkhead box protein O1 (FOXO1), which subsequently upregulates the transcription of antioxidant enzymes, thereby enhancing renal ROS scavenging (Xu et al., 2016). Furthermore, peroxisome proliferator-activated receptor γ coactivator 1-α (PGC-1α), a regulator of mitochondrial biogenesis and respiratory chain complex formation (Islam et al., 2018; Qian et al., 2019), is upregulated in response to activated FOXO1, thereby diminishing mitochondrial oxidative injury. Through the SIRT1–FOXO1–PGC-1α axis, puerarin attenuates oxidative stress-mediated podocyte injury and glomerular structural disruption (Xu et al., 2016).

NADPH oxidase 4 (NOX4) is a member of the NOX family of transmembrane oxidoreductases that generates ROS, primarily H_2_O_2_, by transferring electrons from NADPH to molecular oxygen (Vermot et al., 2021). NOX4 is primarily localized to the endoplasmic reticulum (ER) and mitochondria, resulting in substantial ROS accumulation (Matsushima and Sadoshima, 2022; Peng et al., 2023; Ochoa et al., 2018). Compared with the STZ model, STZ-induced DN in endothelial nitric oxide synthase knockout (eNOS^−/−^) mice elicits more severe renal injury, thereby more closely recapitulating human DN (Li et al., 2017; Verón et al., 2022). In eNOS^−/−^ mice, puerarin activates SIRT1 to suppress nuclear factor-κB (NF-κB) acetylation, thereby downregulating NOX4, attenuating oxidative stress in podocytes, preserving glomerular structure, and ultimately reducing proteinuria to ameliorate renal injury (Li et al., 2017).

Moreover, cisplatin-induced nephrotoxicity in rats is characterized by NOX activation, mitochondrial dysfunction, lipid peroxidation, and DNA fragmentation in renal tubular cells (Nagwani and Tripathi, 2010). The receptor for receptors of advanced glycation end products (RAGE) is a multiligand transmembrane protein that, upon binding to advanced glycation end products (AGEs), activates intracellular NOX4 (Guan et al., 2022), thereby increasing ROS generation and exacerbating oxidative stress (Koulis et al., 2015). Puerarin disrupts the AGE–RAGE interaction, restores SOD and GSH-Px activity, and alleviates high glucose-induced oxidative stress in glomerular MCs (Wang and Li, 2024; Zhang Z. T. et al., 2021). Additionally, puerarin can inhibit NOX4 by upregulating protein kinase C beta type (PKCβ) expression (Wang and Li, 2024).

Nuclear factor erythroid 2-related factor 2 (Nrf2) is the transcriptional regulator of cellular antioxidant responses and orchestrates the expression levels of genes involved in oxidative stress defence (Ngo and Duennwald, 2022; Xiang et al., 2022). Physiologically, Nrf2 binds to Kelch-like ECH-associated protein 1 (KEAP1) and is inactive in the cytoplasm and is degraded through the ubiquitination pathway, thereby maintaining low levels of Nrf2 in the nucleus (Baird and Yamamoto, 2020; Nezu et al., 2017). Puerarin induces conformational changes in KEAP1, liberating Nrf2 from its complex. Nrf2 is translocated to the nucleus, binds to antioxidant response elements (AREs), and initiates the transcription of antioxidant genes, including haem oxygenase-1 (HO-1), resulting in reduced ROS accumulation and protection of podocytes in DN mice. This mechanism restores podocin expression, maintains glomerular filtration barrier (GFB) integrity, and reduces proteinuria (Gao et al., 2023; Wang J. S. et al., 2025; Wang J. C. et al., 2019). Puerarin modulates Nrf2 activity by upregulating the expression of Parkinson disease protein 7 (DJ-1) and 5′-AMP-activated protein kinase (AMPK) (Xue S. et al., 2025).

In summary, puerarin alleviates renal oxidative stress through coordinated regulation of multiple signaling pathways, including SIRT1, FOXO1, NF-κB, and Nrf2 (Figure 1). Antioxidative activity is supported by substantial experimental evidence and appears to be among the most extensively investigated renoprotective mechanisms of puerarin. However, most studies have focused on individual oxidative stress-related pathways under specific experimental conditions, while their relative contributions and interactions with other pathological processes remain unclear. As illustrated in Figure 1, oxidative stress not only directly damages renal cells but also acts as an upstream driver of inflammation and fibrosis, suggesting that its modulation may influence multiple downstream pathological processes.

### 2.2 Anti-inflammation

Persistent inflammation contributes to glomerular structural injury, tubular fibrosis, and immune dysregulation, ultimately leading to irreversible loss of renal function and progression to ESRD (Kadatane et al., 2023; Stenvinkel et al., 2021). In experimental models of DN, AKI, and nephrolithiasis, puerarin has been reported to suppress the release of inflammatory factors and inhibit renal inflammatory responses, including proinflammatory cytokines, such as interleukin (IL)-6, IL-1β, and IL-8, tumour necrosis factor-α (TNF-α), and chemokine monocyte chemoattractant protein-1 (MCP-1) (Xu et al., 2024; Li et al., 2024; Hu et al., 2024; Zhou L. B. et al., 2024). Puerarin effectively inhibits inflammation, alleviates kidney injury, and prevents the occurrence and progression of kidney diseases (see Figure 1).

In lipopolysaccharide-induced and unilateral ureteral obstruction (UUO)-induced AKI mouse models, puerarin ameliorates renal injury and inflammation by interrupting the interaction between toll-like receptor 4 (TLR4) and the myeloid differentiation primary response protein MyD88 (MyD88) through direct binding to the TIR domain of MyD88, thereby reducing the release of inflammatory cytokines (Hu et al., 2024). MicroRNAs (miRNAs) are a class of endogenously derived, noncoding short-chain RNAs that regulate cellular activity and gene expression and have been implicated as important microscopic factors in kidney disease pathogenesis and therapy (Mahtal et al., 2022). In a STZ-induced DN rat model, puerarin upregulates miRNA-140-5p, which targets and inhibits the binding of TLR4 to MyD88, thereby suppressing inflammatory responses, protecting TECs from injury, and reducing fibrosis and apoptosis (Xu Y. M. et al., 2020). The TLR4–MyD88 axis can activate the c-Jun N-terminal kinase (JNK)/FOXO1 pathway, driving M1 macrophage polarization; by blocking this interaction, puerarin can inhibit the proinflammatory M1 macrophage phenotype (Hu et al., 2024). As a major component of the Fuling-Zexie formula, puerarin may attenuate the activity of the tyrosine protein kinase JAK2 (JAK2)/signal transducer and activator of transcription 3 (STAT3) signaling pathway via TLR4, thereby suppressing the expression levels of proinflammatory cytokines and further supporting its anti-inflammatory role in renal disease (Lu et al., 2024).

NF-κB plays a central regulatory role in renal inflammatory signalling networks (Mahtal et al., 2022). Puerarin suppresses the activation of the LRR and PYD domain-containing protein 3 (NLRP3) inflammasome, which is triggered by NF-κB p65, by suppressing the interaction between TLR4 and MyD88 (Lu et al., 2024; Guan et al., 2020; Gao et al., 2024). This inhibition blocks the nuclear translocation of NF-κB, reducing the expression levels of proinflammatory cytokines and ameliorating cisplatin-induced AKI (Xu Y. M. et al., 2020; Ma et al., 2017). In UUO-induced CKD models, puerarin protects kidneys by inhibiting the NF-κB p65/STAT3 signalling pathway and thus further dampening inflammation (Wang J. Y. et al., 2021). Notably, puerarin can have synergistic effects when it is combined with other compounds. In db/db mice, the combined administration of puerarin and arctigenin strongly inhibited NF-κB activity through two complementary mechanisms. Arctigenin reduces the phosphorylation of the NF-κB p65 subunit by activating protein phosphatase 2A (PP2A), whereas puerarin decreases the acetylation of NF-κB p65 by promoting SIRT1 activation. This cooperative inhibition of NF-κB results in decreased expression of proinflammatory cytokines in podocytes, thereby ameliorating glomerular hypertrophy (Li et al., 2024).

The phosphatidylinositol 3-kinase (PI3K)/RAC-alpha serine/threonine-protein kinase (AKT) pathway is another mechanism through which puerarin exerts its anti-inflammatory effects. In a calcium oxalate (CaOx) kidney stone model, puerarin activated PI3K/AKT signalling, thereby attenuating TEC inflammation and reducing IL-1β, IL-6, and TNF-α levels (Xu et al., 2024).

Puerarin primarily regulates renal inflammation by targeting the TLR4 and NF-κB pathways, persistent inflammation further amplifies oxidative injury and promotes renal fibrosis, highlighting the close crosstalk among these pathological processes. However, renal inflammation involves complex interactions among multiple cell types and cytokine networks, whereas current studies mainly focus on canonical inflammatory pathways. Whether puerarin exerts broader immunomodulatory effects within the renal microenvironment remains unclear.

### 2.3 Antifibrosis

Renal fibrosis is a hallmark of progressive kidney disease characterized by excessive deposition of extracellular matrix (ECM), tubular injury, and infiltration of inflammatory cells. These pathological changes lead to deterioration of renal function and may ultimately progress to ESRD (Li et al., 2022). Puerarin ameliorates renal fibrosis mainly by regulating the expression of transforming growth factor-β (TGF-β)/Smad family member (Smad) (Figure 1).

In STZ-induced DN models, puerarin attenuates renal injury by inhibiting TGF-β1-mediated Smad2 activation and reducing the expression levels of CCN family member 2 (CTGF) and fibronectin (FN). This intervention limits excessive glomerular ECM accumulation and thereby alleviates glomerulosclerosis and interstitial fibrosis (She et al., 2014). In high glucose-induced MC injury, liposome-encapsulated puerarin suppresses TGF-β1 overexpression and blocks the nuclear translocation of Smad2/3, thus inhibiting MC proliferation and profibrotic signalling activation and ultimately interfering with DN progression (Barro et al., 2021). In chronic kidney injury induced by UUO, puerarin downregulates the expression levels of profibrotic factors such as TGF-β1, Smad3, and p-Smad3 and upregulates the expression of Smad7 to impede fibrotic signalling. This phenomenon results in reduced expression of α-smooth muscle actin (α-SMA), vimentin, and type I collagen (COL I), inhibits ECM accumulation, and protects renal function (Wang J. Y. et al., 2021).

In addition to affecting the TGF-β/Smad axis, puerarin influences renal fibrosis via the regulation of iron metabolism and ferroptosis pathways. In high-fat diet-fed and STZ-induced diabetic rats, puerarin restored iron homeostasis and inhibited ferroptosis, thereby decreasing ECM accumulation and collagen deposition. In high glucose-induced glomerular MCs, puerarin downregulates the expression of collagen (COL I and COL IV), FN, α-SMA, and TGF-β, attenuating fibrosis (Hou et al., 2024). In ischaemia‒reperfusion (I/R)-induced interstitial fibrosis, puerarin modulates ferroptosis through the TLR4/NOX4 pathway and mitigates oxidative stress, thereby reducing collagen deposition and α-SMA expression while ameliorating fibrosis (Jian et al., 2023).

The TLR4/NOX4 axis is closely associated with oxidative stress, which is a key driver of renal fibrosis. UUO induces renal fibrosis by exacerbating oxidative stress responses and upregulating NOX4 expression, resulting in pronounced deposition of interstitial collagen, FN, and ECM (Zhou et al., 2017). In STZ-induced diabetic rats, puerarin attenuates oxidative stress, decreases MMP9 expression, and ameliorates podocyte foot process effacement and glomerular mesangial expansion associated with fibrosis (Zhong et al., 2014).

Overall, puerarin attenuates renal fibrosis through coordinated regulation of the TGF-β/Smad pathway, ferroptosis, and oxidative stress. As illustrated in Figure 1, renal fibrosis is the cumulative consequence of sustained oxidative stress and chronic inflammation, indicating that antifibrotic activity represents a major downstream mechanism of puerarin-mediated renoprotection. Although supported by substantial experimental evidence, most studies focus on individual profibrotic pathways, while the interactions among different renal cell populations during fibrosis progression remain poorly understood.

### 2.4 Inhibition of apoptosis

**FIGURE 2:**
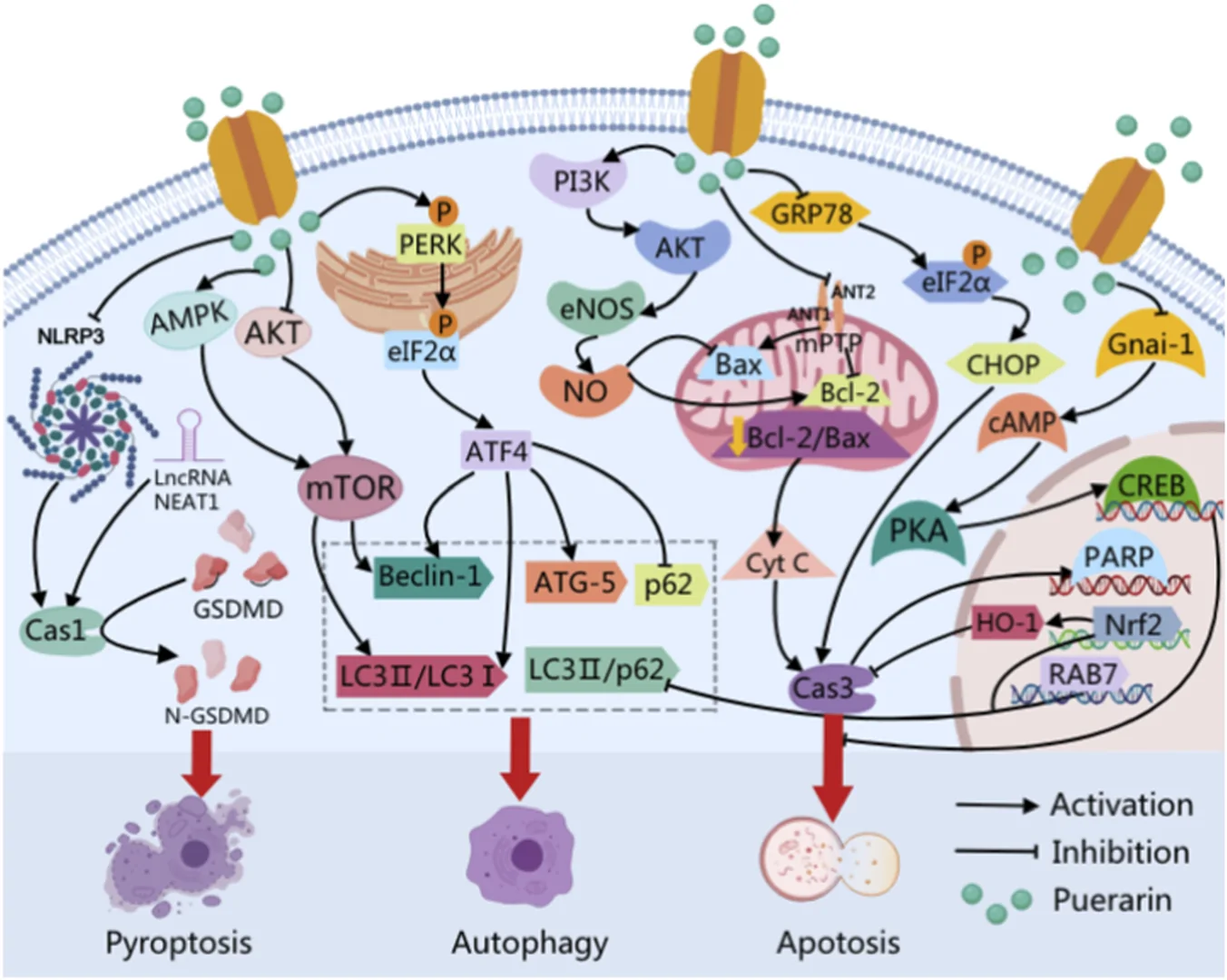
Puerarin regulates apoptosis, autophagy, and pyroptosis in kidney diseases. AKT, RAC-alpha serine/threonine-protein kinase; AMPK, 5′-AMP-activated protein kinase; ANT, Adenine nucleotide translocator; ATF4, Cyclic AMP-dependent transcription factor ATF-4; ATG-5, Autophagy protein 5; Bax, Apoptosis regulator BAX; Bcl-2, Apoptosis regulator Bcl-2; Cas, Caspase; CREB, Cyclic AMP-responsive element-binding protein; CHOP, DNA damage-inducible transcript 3 protein; Cyt c, Cytochrome c; eIF2α, Eukaryotic translation initiation factor 2A; eNOS, Nitric oxide synthase; Gnai1, Guanine nucleotide-binding protein G(i) subunit alpha-1; GRP78, Endoplasmic reticulum chaperone BiP; GSDMD, Gasdermin D; HO-1, Heme oxygenase-1; LC 3, Light chain 3; lncRNA, Long noncoding RNA; mPTP, Mitochondrial permeability transition pore; mTOR, Mechanistic target of rapamycin; Nrf2, Nuclear factor erythroid 2-related factor 2; PARP, Poly (ADP-Ribose) polymerase; PERK, Protein kinase R-like endoplasmic reticulum kinase; PI3K, Phosphatidylinositol 3-kinase; PKA, Protein kinase A; and RAB7, Ras-related protein Rab-7a.

Apoptosis is a form of programmed cell death in multicellular organisms that plays a critical role in maintaining renal homeostasis. Various pathological stimuli, including exposure to lead or cadmium, renal I/R, CaOx deposition, and hyperglycaemia, can induce mitochondrial dysfunction, thereby triggering apoptosis in renal cells (Wang J. S. et al., 2025; Song et al., 2016; Liu et al., 2012; Zhu et al., 2022). Mitochondrial dysfunction is recognized as a pivotal initiator of apoptosis (Jaiswal et al., 2015). Puerarin has been shown to inhibit renal apoptosis by restoring mitochondrial function (Figure 2).

Oxidative stress contributes to mitochondrial dysfunction in renal cells. Puerarin effectively reduces ROS accumulation in renal cells, restores GSH levels, decreases MDA levels, and rebalances the redox system (Wang J. S. et al., 2025; Liu et al., 2012). During cadmium-induced apoptosis of proximal renal tubular cells, puerarin regulates the expression levels of adenine nucleotide translocator (ANT) 1 and ANT2, inhibits the opening of the mitochondrial permeability transition pore (mPTP), maintains the potential of the mitochondrial membrane, alleviates depolarization of the mitochondria, and prevents the release of cytochrome c (Cyt c) from the cytosol. This sequence ultimately blocks the activation of caspase 3 and poly (ADP–ribose) polymerase (PARP) cleavage. Furthermore, puerarin upregulates the expression of the apoptosis regulator Bcl-2 (Bcl-2), downregulates the expression of the apoptosis regulator BAX (Bax), and increases the Bcl-2/Bax ratio, collectively inhibiting cadmium-induced apoptosis and providing a basis for preventing cadmium-induced nephrotoxicity (Song et al., 2016).

Similar protective mechanisms are observed in lead- and CaOx-induced renal tubular apoptosis. Through activation of the PI3K/AKT/eNOS signalling pathway, puerarin restores the balance between Bcl-2 and Bax, suppresses Cyt c release, and decreases caspase 3 activity, thereby exerting robust antiapoptotic effects (Xu et al., 2024; Liu et al., 2012).

In addition to providing mitochondrial protection, puerarin modulates apoptosis through several key proteins, including guanine nucleotide-binding protein G(i) subunit alpha-1 (Gnai1), Nrf2, and cyclic AMP-responsive element-binding protein (CREB). In db/db mouse models of type 2 diabetes, puerarin binds directly to and inhibits Gnai1, activating the cAMP/PKA/CREB axis. This action suppresses high glucose-induced podocyte apoptosis and reduces glomerular injury (Zhu et al., 2022). In a rat model of renal I/R injury, puerarin activated the Nrf2/HO-1 pathway and decreased caspase 3 expression (Wang J. S. et al., 2025); in addition, it downregulated the expression of the endoplasmic reticulum chaperone BiP (GRP78), eukaryotic translation initiation factor 2A (eIF2α), DNA damage-inducible transcript 3 protein (CHOP), and caspase 3, thus effectively inhibiting both apoptosis and endoplasmic reticulum stress (ERS) (Wang J. S. et al., 2025; Lan et al., 2025; Gundu et al., 2022). In STZ-induced DN rats, treatment with Pueraria tuberosa extract attenuated renal apoptosis, as evidenced by reduced expression of Bax, active caspase-3, and cleaved PARP-1, together with increased expression of the Bcl-2 (Shukla et al., 2018).

Taken together, these findings indicate that puerarin inhibits renal apoptosis by modulating multiple signalling pathways, restoring mitochondrial integrity, and mitigating oxidative stress. These multifaceted protective actions underscore the therapeutic potential of puerarin in preventing and treating diverse forms of kidney injury and nephrotoxicity, thus warranting further clinical investigation.

### 2.5 Autophagy regulation

Autophagy is a self-protective mechanism that ensures intracellular and extracellular homeostasis (Gómez-Virgilio et al., 2022). Autophagy facilitates the removal of damaged organelles and protein aggregates in renal cells, maintaining cellular integrity. However, pathological states can disrupt autophagic function, contributing to kidney disease progression (Rechter et al., 2016; Lin et al., 2019; Tang et al., 2020; Choi, 2020). Notably, puerarin exerts a bidirectional regulatory effect on autophagy, activating insufficient autophagy or suppressing excessive autophagic activity depending on the pathological context of the kidney (Jing G. H. et al., 2022; Li et al., 2020; Liu G. et al., 2020).

In DN, puerarin enhances autophagy through multiple signalling pathways, thereby preserving glomerular structure and function. Specifically, puerarin activates the protein kinase R-like ER kinase (PERK)/eIF2α/cyclic AMP-dependent transcription factor ATF-4 (ATF4) axis, upregulating the expression levels of autophagy-related proteins, including Beclin-1, light chain 3II (LC3II), and autophagy protein 5 (Atg5), and downregulating the expression of p62, thereby promoting podocyte autophagy. This phenomenon, in turn, mitigates ERS and elevates the levels of nephrin, podocin, and podocalyxin in podocytes, sustaining the integrity of the GFB (Xu X. H. et al., 2020). Additionally, puerarin activates AMPK activity and inhibits mechanistic target of rapamycin (mTOR) signalling, further upregulating Beclin-1 expression and the LC3II/LC3I ratio and facilitating autophagic flux in the kidney. Through enhanced clearance of damaged organelles, these pathways collectively maintain podocyte structure and function and ameliorate DN-related kidney injury (Su et al., 2023).

With respect to TEC injury induced by CaOx crystals, puerarin exerts a protective effect by modulating autophagy-related pathways to inhibit excessive autophagy. It upregulates SIRT1 expression, thereby suppressing downstream AKT activity and increasing p38 MAPK activity. This difference effectively inhibits excessive CaOx crystal-induced autophagy in TECs, preventing autophagy-related cellular damage and thus protecting the kidney (Jing G. H. et al., 2022).

Furthermore, in cadmium-induced renal tubular injury, puerarin restored cellular homeostasis by modulating autophagic flux. It inhibits Nrf2-mediated autophagy while restoring Ras-related protein Rab-7a (RAB7)-mediated autophagic flux, as reflected by reduced LC3-II and p62 protein levels and alleviated ERS. Collectively, these actions mitigate cadmium-induced renal injury (Liu G. et al., 2020). These findings underscore the close interplay between autophagy regulation and ERS reduction, highlighting the precise modulatory capacity of puerarin.

By targeting diverse autophagy-related pathways, puerarin can increase autophagy to promote the clearance of damaged components, inhibit excessive autophagic activity to prevent cell injury, or modulate autophagic flux to restore homeostasis (Figure 2). Through these mechanisms, puerarin demonstrates both the versatility and specificity required for renoprotection across a spectrum of kidney injuries.

### 2.6 Regulation of pyroptosis

Pyroptosis is an inflammatory form of programmed cell death, with caspase-1 serving as a pivotal mediator in the kidney (Luo et al., 2023; Zhang K.-J. et al., 2021). Pyroptosis acts as both a pathological mechanism and a potential therapeutic target in kidney diseases (Zhang K.-J. et al., 2021; Cuevas and Pelegrín, 2021). Puerarin can exert protective effects in various models of renal injury through the modulation of caspase-1 (Figure 2).

In STZ-induced DN, high glucose levels activate the podocyte NLRP3 inflammasome, triggering caspase-1 activation and subsequent cleavage of gasdermin D (GSDMD). This sequence increases cell membrane permeability and initiates podocyte pyroptosis (Chen Q. Y. et al., 2024). Puerarin can reduce the activation of podocyte pyroptosis under high glucose stimulation by downregulating SIRT1 expression and inhibiting the activation of the NLRP3/caspase-1/GSDMD pathway. Consequently, puerarin suppresses renal expression of the proinflammatory cytokines IL-1β and IL-18 and restores the expression levels of podocyte functional proteins, such as nephrin, podocin, and podocalyxin (Wang L. et al., 2025). This antipyroptotic mechanism is similarly observed in renal I/R injury, in which puerarin suppresses the NLRP3/caspase-1/GSDMD axis, resulting in anti-inflammatory, antioxidant, and renoprotective effects (Wang et al., 2023).

In chronic renal failure (CRF) models, the regulatory effect of puerarin on the caspase-1 pathway is evident. An adenine-enriched diet can induce CRF in SD rats, leading to reduced renal expression of miR-342-3p. Puerarin treatment upregulates the expression of miR-342-3p, which, in turn, inhibits TEC pyroptosis by suppressing the activity of the TGF-β/Smad signalling pathway (Yang et al., 2023). Furthermore, puerarin downregulates the expression of the long noncoding RNA (lncRNA) NEAT1, thereby inhibiting caspase-1/GSDMD-N-mediated pyroptosis, reducing the release of IL-1β and IL-18, and ameliorating TEC injury in CRF (Yang J. et al., 2024).

Collectively, these studies highlight the central involvement of the caspase-1 pathway in the puerarin-mediated regulation of renal pyroptosis, with key modulators such as SIRT1, miR-342-3p, and lncRNA NEAT1 contributing to these effects (Figure 2). Further elucidation of the regulatory mechanisms of puerarin in pyroptosis may facilitate the development of novel strategies for renal protection.

Compared with oxidative stress and inflammation, the regulation of autophagy and pyroptosis by puerarin has received relatively less experimental attention. Although several signaling pathways have been implicated, the available evidence remains fragmented, making it difficult to define their relative contributions to renal protection. As illustrated in Figure 2, apoptosis, autophagy, and pyroptosis constitute an interconnected network of regulated cell death, suggesting that puerarin-mediated renoprotection is more likely achieved through coordinated modulation of these pathways rather than regulation of any single process.

### 2.7 Modulation of lipid metabolism

Abnormal lipid metabolism is increasingly recognized as a critical contributor to renal pathology. Under physiological conditions, the kidney contains relatively low levels of lipids; however, lipid accumulation is evident in the early stages of CKD and actively drives disease progression (Baek et al., 2021). Excessive lipid deposition within the kidney can injure podocytes, endothelial cells, TECs, and macrophages, ultimately impairing renal architecture and promoting inflammation and fibrosis (Gai et al., 2019; Opazo-Ríos et al., 2020). Notably, both puerarin and canagliflozin attenuate the onset and progression of DN by inhibiting CD36/adipophilin-mediated lipid uptake, activating peroxisome proliferator activated receptor α (PPARα) to increase fatty acid oxidation, and reducing renal lipid accumulation, which collectively mitigate oxidative stress and inflammatory responses (Zhu et al., 2023).

The mechanisms by which puerarin affects lipid metabolism include suppressing lipoprotein lipolysis, decreasing fatty acid (FA) uptake, promoting FA degradation, and inhibiting the synthesis of FAs and cholesterol (Jing X. et al., 2022). PPARs are ligand-activated nuclear receptors that play key roles in lipid homeostasis; fibrates, which are widely used as hypolipidaemic agents in clinical practice, activate PPARs to govern both the synthesis and oxidation of FAs (Kamijo, 2018; Liu G. et al., 2023). Long-term use of statins increases renal lipid uptake and inhibits fatty acid oxidation, thereby exacerbating ectopic fat deposition in the kidneys of diabetic mice (Huang et al., 2023). Given these limitations of current pharmacological interventions, the identification and development of natural bioactive compounds, such as puerarin, with low toxicity and minimal side effects for the management of renal lipid metabolic disorders has emerged as a central focus of contemporary nephrology research (Zhang et al., 2025; Rong et al., 2022; Yang M. et al., 2024).

Regulation of lipid metabolism has emerged as a potential mechanism underlying the renoprotective effects of puerarin. However, current evidence is largely confined to DN, and whether similar metabolic regulation occurs in other kidney diseases remains uncertain.

### 2.8 Promotion of renal clearance

As a vital metabolic organ, the kidneys play an essential role in maintaining systemic homeostasis. Recent studies suggest that puerarin ameliorates drug-induced and metabolic kidney injury by modulating renal transporters and associated signalling pathways (Lu et al., 2024; Liu et al., 2014; Liu Q. et al., 2018; Xu et al., 2022).

The renoprotective effect of puerarin in methotrexate (MTX)-induced nephrotoxicity is closely linked to the regulation of renal transporter solute carrier family 22 member 6 (OAT1) and solute carrier family 22 member 8 (OAT3). Puerarin can directly upregulate OATs in the kidney, thereby facilitating the excretion of MTX and its metabolites, reducing plasma MTX concentrations, and increasing urinary elimination (Liu et al., 2014; Liu Q. et al., 2018). Additionally, puerarin activates B-cell lymphoma 6 protein homologue (BCL6), which suppresses the NF-κB-mediated inflammatory pathway and decreases prostaglandin E2 (PGE2) levels, further increasing OAT1/3 expression. This phenomenon establishes a BCL6-OAT1/3 signalling axis that synergistically mitigates MTX-induced nephrotoxicity (Liu Q. et al., 2018).

Puerarin also protects against hyperuricaemia-induced renal injury. It alleviates tubular swelling, reduces inflammatory cell infiltration, and glomerular atrophy, and effectively improves renal uric acid excretion function (Xu et al., 2022). The underlying mechanism involves the regulation of uric acid transport-related proteins: puerarin increases the expression of ATP-binding cassette subfamily G member 2 (ABCG2), thus promoting the active secretion of uric acid from renal tubular cells into the urine; concurrently, it downregulates glucose transporter 9 (GLUT9), reducing the reabsorption of uric acid from the tubular lumen back into the circulation. These synergistic actions help restore uric acid metabolic balance and protect against hyperuricaemia-induced renal injury (Lu et al., 2024).

Despite the encouraging progress summarized in this review, several challenges remain. Most mechanistic evidence is derived from experimental models, while clinical validation remains limited. In addition, differences in disease models and experimental designs hinder direct comparison across studies, and the relative importance of individual signaling pathways during different stages of kidney disease remains unclear. Addressing these issues will be essential for facilitating the clinical translation of puerarin-based therapeutics.

## 3 Prospective strategies for kidney-targeted delivery of puerarin

The rational design of kidney-targeted puerarin nanomedicines should consider not only the characteristics of the carrier but also the physicochemical and pharmacokinetic properties of puerarin itself. Owing to its poor aqueous solubility, rapid systemic clearance, and limited oral bioavailability, different nanocarrier platforms may offer distinct advantages in improving drug stability, prolonging circulation time, enhancing renal biodistribution, and achieving controlled release. Therefore, the selection and engineering of nanocarriers should be tailored to the specific delivery requirements of puerarin rather than following a universal formulation strategy.

Advances in nanotechnology have enabled the development of diverse drug-delivery platforms, including nanoparticles, hydrogels, liposomes, micelles, dendrimers, and chitosan-based carriers (Iacob et al., 2021). The application potential of nanocarriers strongly depends on parameters such as their size, shape, hydrophobicity, and surface properties. When the particle size of the carrier reaches the nanoscale, its specific surface area is significantly larger than that of traditional microscale materials, thus enhancing the potential for interaction between the drug and the biological system (Zhao et al., 2019; Cai et al., 2021; Bouchoucha et al., 2016; Kim et al., 2024). The kidney consists of approximately one million nephrons, including glomeruli with a filtration function and renal tubules with a reabsorption function, featuring a complex structure (Sakai, 2017). This organ plays a central role in regulating the body’s water-electrolyte balance, excreting metabolic waste, and adjusting acid-base balance and blood pressure. The structures and processes related to these physiological functions provide potential targets for the treatment of kidney diseases (Imenez Silva and Mohebbi, 2022).

Puerarin has demonstrated anti-inflammatory, antioxidant, and antifibrotic activities in various kidney disease models. However, its clinical translation is hindered by poor aqueous solubility, low oral bioavailability, rapid metabolism, and a lack of intrinsic renal-targeting capability, all of which limit its therapeutic efficacy in renal disorders (Cheng et al., 2024). Nanotechnology-based delivery systems offer a promising strategy to overcome these limitations by improving drug solubility, prolonging circulation time, enhancing tissue accumulation, and enabling targeted delivery. Nevertheless, direct evidence for kidney-targeted puerarin nanomedicines remains limited, as most currently reported puerarin-loaded nanocarriers have been evaluated in non-renal disease models. Therefore, the following sections summarize the principles of passive and active renal targeting that may provide a theoretical framework for the future development of kidney-targeted puerarin nanodelivery systems.

### 3.1 Passive targeting

The passive targeting of nanomedicines to the kidney relies on the carrier passing through the GFB and entering the renal tubule; this mainly depends on the physicochemical properties of the nanoparticles, including their particle size, surface charge, and shape (Oroojalian et al., 2020).

#### 3.1.1 Particle size regulation

**FIGURE 3:**
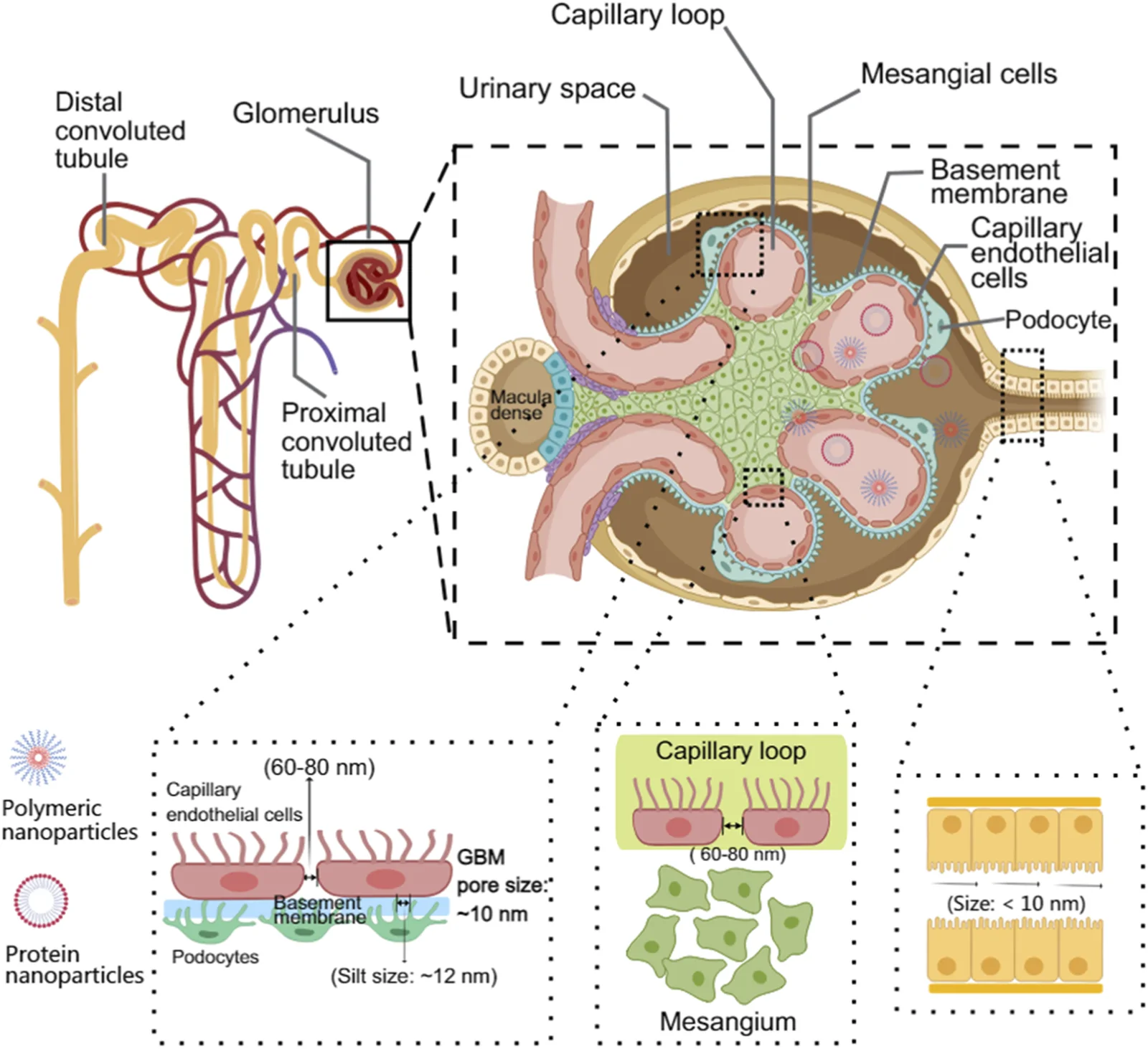
Passive targeting of puerarin nanoparticles to the kidneys. Capillary endothelial cells, which are separated from podocytes by the basement membrane, together constitute the GFB. Nanoparticles with ∼12 nm can traverse the GFB and enter the renal tubular lumen, supporting glomerular filtration and tubular delivery. Nanoparticles ranging from 10 to 200 nm may preferentially accumulate within glomerular structures. Among them, particles of 60–80 nm can pass through endothelial fenestrations and undergo mesangial retention, whereas particles of 80–100 nm are less efficiently filtered and may remain within glomerular compartments. Small polymeric carriers with diameters of 5–30 nm have also been reported to cross the GFB and accumulate in podocytes, suggesting potential applications for podocyte-targeted delivery.

The GFB is composed of endothelial cells, basement membrane, and podocytes. The fenestrae of endothelial cells have a diameter of 60–80 nm, and the slit diaphragms between podocyte foot processes have a diameter of 12–22 nm, forming a physical barrier for drug delivery (Figure 3). Research has indicated that particles with ∼12 nm can readily pass through the GFB and enter the renal tubular lumen with minimal obstruction (Aird, 2007; Gagliardini et al., 2010). Nanoparticles with diameters ranging from 10 to 200 nm may preferentially accumulate within glomerular structures, including podocytes and mesangial regions. Among them, particles with a diameter of 80–100 nm are more difficult to filter by the glomerulus (Huang et al., 2020; Huang Y. et al., 2021). For example, owing to their size advantage, star-shaped polyethylene glycol (PEG) polymers with a particle size of 5–30 nm can cross the GFB and specifically accumulate in podocytes. Such carriers have been reported to cross the GFB and accumulate in podocytes, facilitating the delivery of therapeutic agents (Bruni et al., 2017). In addition, under pathological conditions, the gaps in the GFB increase (Liu G. W. et al., 2020). For instance, in Alport syndrome, the collagen structure of the basement membrane is damaged, leading to increased porosity and allowing larger-diameter nanoparticles to pass through (Funk et al., 2018; Cosgrove and Liu, 2016); this may provide additional opportunities for passive targeting. Collectively, particle size may represent an important consideration for future kidney-targeted puerarin delivery.

#### 3.1.2 Surface charge modification

The GFB is rich in negatively charged proteoglycans, which strongly repel negatively charged particles (Zhao et al., 2021a; Huang X. et al., 2021). In contrast, nanoparticles with a positively charged surface have been reported to exhibit enhanced interactions and accumulate in renal tubules, thus prolonging the drug half-life. Therefore, in the design of puerarin nanocarriers, materials with a mildly positively charged surface may represent a potential strategy for future renal delivery of puerarin, potentially influencing renal distribution and retention after filtration. However, high-density and strongly positive surface-charge modification should be avoided to prevent cytotoxicity and nonspecific protein adsorption, ensuring the biosafety of the carrier (Huang et al., 2020).

#### 3.1.3 Particle shape design

The shape of the particles significantly influences the *in vivo* behaviour of nanocarriers. Research has shown that the *in vivo* clearance rate of spherical nanoparticles is faster than that of nonspherical nanoparticles (such as oblate and rod-shaped nanoparticles). The latter often exhibit prolonged circulation and tissue residence times, which may contribute to altered interactions with biological barriers and tissue distribution profiles (Zhang Z. et al., 2019). Even when one dimension exceeds the typical filtration threshold, rod-shaped or cylindrical nanoparticles have been reported to traverse the GFB under certain conditions (Lee et al., 2021). In addition, some studies have suggested that nonspherical particles may reduce macrophage uptake, decrease the clearance of the carrier by the immune system, and facilitate the accumulation of drugs in renal tissues (Sakamoto et al., 2024). Collectively, particle shape may influence renal biodistribution and targeting efficiency. However, its applicability to puerarin-loaded nanocarriers requires further experimental validation in renal disease models.

### 3.2 Active targeting

The active targeting of nanomedicines to the kidney may be achieved by introducing specific functional groups or bioactive molecules, such as receptor ligands of TECs and glomerular-specific proteins, onto the surface of the carrier. This strategy may enhance the interaction between nanocarriers and specific renal cell populations, potentially improving site-selective drug delivery (Cheng et al., 2024; Huang et al., 2020; Tietjen et al., 2018). These targeting molecules include mainly polypeptides and antibodies, with antibodies being the most widely used (Wang et al., 2017a). However, antibody modification significantly increases the particle diameter and hinders glomerular filtration (Chen et al., 2018). In contrast, peptide ligands, as targeting molecules, can maintain targeting specificity without significantly increasing the particle volume (Wang et al., 2017a). If kidney-targeted puerarin nanocarriers are developed, ligand selection could potentially be guided by the receptor profiles of different renal cell populations (Colombo et al., 2016).

The mechanistic studies summarized in Table 1 indicate that different renal pathological processes involve distinct effector cell populations, particularly podocytes, MCs, and TECs. These cell populations represent potential targets for kidney-directed drug delivery. Therefore, active-targeting strategies may be developed according to disease-associated cellular targets and receptors, providing a rationale for the design of cell-specific puerarin delivery systems.

#### 3.2.1 Targeting podocytes

**FIGURE 4:**
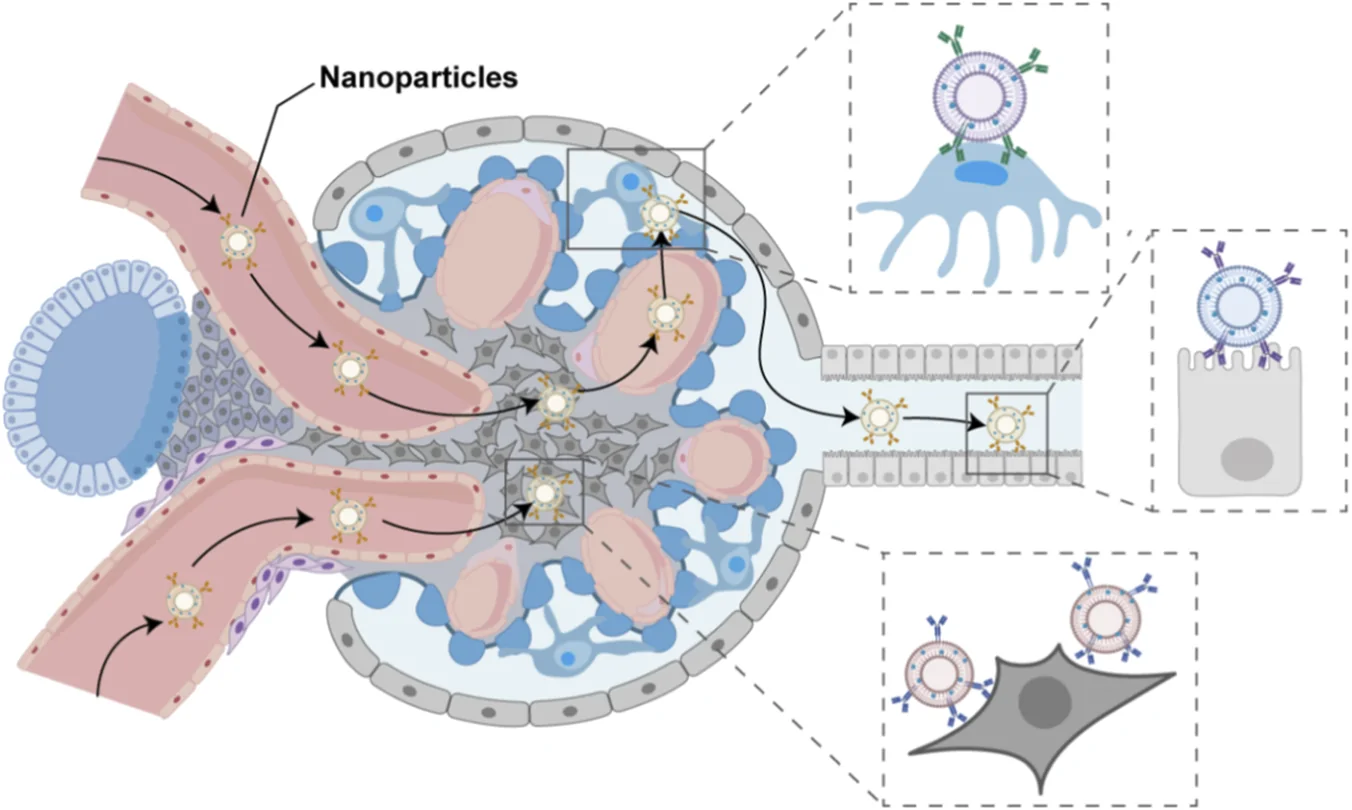
Active targeting of puerarin nanoparticles to the kidneys. Various puerarin nanoparticles are designed with different ligands targeting the surface of renal cells, aiming to achieve the specific binding of puerarin nanoparticles to renal cells.

There are various specific receptors on the surface of podocytes, such as nephrin and podocin (Morales-Betanzos et al., 2024). Although research on specific ligands for these receptors is still ongoing, polypeptide sequences that can specifically bind to the receptors on the surface of podocytes can be obtained through screening (Huang C. et al., 2021) (Figure 4). If incorporated into future puerarin nanocarriers, such polypeptides may facilitate receptor-mediated podocyte targeting and could potentially improve local drug exposure within podocytes. These findings support the feasibility of podocyte-targeted puerarin delivery strategies. In addition to the polypeptide-targeting modification strategy, various nanocarriers have achieved efficient targeted delivery to podocytes through different mechanisms, which may provide useful insights for the future development of puerarin nanocarriers. For example, antibody-targeted liposomes can specifically recognize vascular cell adhesion molecule-1 (VCAM-1) molecules highly expressed on the surface of podocytes in an inflammatory microenvironment, achieving the precise delivery of drugs such as rapamycin and significantly enhancing therapeutic efficacy (Visweswaran et al., 2015). By utilizing the FcRn receptors highly expressed by podocytes, albumin nanoparticles have been reported to deliver drug complexes such as albumin-methylprednisolone through receptor-mediated endocytosis, increasing podocyte uptake and cytoprotective responses in experimental models (Wu et al., 2017) (Figure 4).

#### 3.2.2 Targeting MCs

MCs specifically express the Thy1.1 antigen on their surface (Tuffin et al., 2005). If puerarin nanocarriers are developed for mesangial targeting, antibody fragments or peptides capable of binding Thy1.1 may represent potential targeting ligands for carrier modification (Figure 4). Such a strategy may facilitate preferential interaction of puerarin-loaded nanocarriers with MCs and increase local drug exposure within the mesangial region. However, its therapeutic value in mesangial cell-associated glomerular disorders remains to be established. In addition, other design strategies for nanocarriers targeting MCs provide important references for the optimization of puerarin delivery systems. For example, α8-integrin-antibody-modified poly (lactic-co-glycolic acid) (PLGA)-liposome hybrid nanoparticles (Zhou et al., 2022) and gold-nanoparticle-immunoliposomes (Fang et al., 2021) can achieve efficient targeted delivery to MCs, improving local drug accumulation and therapeutic responses in experimental models using agents such as dexamethasone and TGF-β1 siRNA. These findings may inform the design of future puerarin nanocarriers.

#### 3.2.3 Targeting TECs

TECs have various receptors on their surface, such as the megalin receptor (Goto et al., 2024) and transferrin receptor. Among them, the megalin receptor can bind to various small-molecule proteins and polypeptides and mediate endocytosis. Currently, nanocarriers targeting the megalin receptor of TECs include micelles (Wang et al., 2018) and polymer nanoparticles (Ordikhani et al., 2020). These findings suggest that megalin may represent a potential target for TEC-directed delivery. However, the applicability of megalin-targeted strategies to puerarin-loaded nanocarriers has not yet been investigated (Figure 4).

## 4 Nanomaterials with potential applications in kidney-targeted delivery of puerarin

Puerarin-loaded nanocarriers have been investigated in a variety of disease models. These include nanoparticles, hydrogels, liposomes, micelles, dendrimers, nanocrystals (NCs), and microemulsions (Table 2). Each material possesses distinct structural and physicochemical characteristics that may influence the delivery behavior of puerarin. Given the complex architecture of the kidney, successful renal drug delivery requires the integration of appropriate carrier systems, therapeutic agents, target cell populations, and targeting strategies (Liu et al., 2021).

**TABLE 2:** Puerarin studies using nanoparticles for disease delivery/treatment.

Number	Disease/Pharmacology	Nanoparticle type	Size (nm)	Zeta Pot (mV)	Load	Animal	References
1	Brain ischaemia–reperfusion injury	Hydroxypropyl beta cyclodextrin-poly (D, L-lactic-co-glycolic acid)	100–300	-	Puerarin	Rat	Tao et al. (2013)
2	Cardiovascular disease	Poly (lactic-co-glycolic acid)	167.1 ± 5.26	−29.88 ± 2.46	Puerarin	Rat	Zhang et al. (2024)
3	Alcohol-induced liver injury	Poly (lactic-co-glycolic acid)	190 ± 2.2	-	Puerarin	Mouse	Han et al. (2021)
4	Cancer	Poly-puerarin	70	-	Paclitaxel	-	Yi et al. (2019)
5	Ocular disease	Carbopol/Methocel E4M gel	-	-	Puerarin	Rabbit	Wu et al. (2007)
6	Liver ischaemia/reperfusion injury	Glycyrrhetinic acid–PEG–PBLA	72	-	Puerarin	Rat	Xiao et al. (2018)
7	Implantation	Poly (lactic-co-glycolic acid)	∼147	+39.95 ± 23.36	Puerarin	Rat	Saraswat et al. (2016)
8	Irritable bowel syndrome with diarrhoea	Alginate/chitosan microspheres	43.3 ± 10.2	-	Puerarin	Rat	Zhang et al. (2022a)
9	-	Chitosan hydrochloride–carboxymethyl chitosan	373.2	+35.2	Puerarin/Resveratrol	-	Wang et al. (2025c)
10	-	AEROPERL®300 Pharma -based solid dispersion	-	-	Puerarin	Rat	Wang et al. (2019b)
11	Osteoarthritis	Chondroitin sulfate microspheres with gallic acid–magnesium MOF	185.8 ± 9.3	-	Puerarin	Rat	He et al. (2025)
12	-	Phenylboronylated chitosan	127.2 ± 0.80/149.8 ± 0.14	-	Puerarin	Rat	Liu et al. (2024)
13	Cerebral ischaemia/reperfusion injury	Poly (butylcyanoacrylate)	201.2	−7.72	Puerarin	Rat	Zhao et al. (2013)
14	Acute myocardial infarction	ANP/TPP–BN–LPNs	98.5 ± 2.9	−19.5 ± 1.9	Baicalin/Puerarin	Rat	Wang et al. (2021c)
15	Lung cancer	PEG–poly (lactic-co-glycolic acid)	81.5 ± 0.6	−7.8 ± 0.3	Puerarin/5FU	-	Zhao et al. (2021b)
16	-	Chitosan	190.32 ± 2.65	+16.5 ± 1.08	Salmon Calcitonin/Puerarin	Rat	Liu et al. (2019)
17	Brain toxicity	Poly (lactic-co-glycolic acid)	190 ± 2.2	-	Puerarin	Mouse	Gu et al. (2023)
18	Ophthalmic diseases	Poly (l-lactide)	188/675	-	Puerarin	-	Chen et al. (2009)
19	Ophthalmic diseases	Hydroxyl-grafting polysiloxane	-	-	Puerarin/Ketoconazole	-	Xu et al. (2014)
20	Spinal cord injury	Poly (lactic-co-glycolic acid)	238–274	−12.6 ± 2.1	Resveratrol/Puerarin	Rat	Chen et al. (2020)
21	-	Poly (lactic-co-glycolic acid)	167.1 ± 5.26	−29.88 ± 2.46	Puerarin	Rat	Zhang et al. (2024)
22	Parkinson’s disease	Six-armed star-shaped poly (lactide-co-glycolic acid)	88.36 ± 1.67	−17.62 ± 2.28	Puerarin	Mouse	Chen et al. (2019)
23	​​Ischaemic stroke​	Polysorbate 85 and H_2_O_2_-sensitive PDAA	32.82 ± 1.32	+16.0 ± 3.1	Puerarin	Rat	Zhang et al. (2023)
24	Atherosclerosis	Cinnamaldehyde polymer	66.62 ± 1.71	16.62	Puerarin	Mouse	Liu et al. (2025)
25	-	Chitosan	190.32 ± 2.65	16.5 ± 1.08	Salmon calcitonin/puerarin	-	Liu et al. (2019)
26	-	Chitosan and sodium tripolyphosphate	∼150	-	Puerarin	Rat	Yan et al. (2020)
27	Wound healing	L-arginine-modified puerarin-derived carbon	151.1	−8.9	Puerarin	Rat	Su et al. (2025)
28	-	Zein/foxtail millet prolamin nanoparticles	106 ± 3.8	∼27	Puerarin/Resveratrol /Diosmetin/Curcumin	-	Chen et al. (2022)
29	-	Phosphorylated hollow zein nanoparticles	138.2–222.4	−27.7 to −25.7	Puerarin	-	Rao et al. (2025)
30	Ophthalmic diseases	Albumin nanoparticles	64.8	−19.6	Puerarin	Rabbit	Hu et al. (2022)
31	Antioxidant	Gold nanoparticles	3.5–50	-	Quercetin/daizeol/puerarin	-	Wang et al. (2007)
32	-	PEGylated mesoporous silica nanoparticles	240	−36	Puerarin	Rat	Liu et al. (2016)
33	Antibacterial/Antimicrobial	Ag2O/Ag nanoparticles	<100	-	Puerarin	-	Liga et al. (2025)
34	Antimicrobial/Angiogenesis	ZnO nanoparticles	200–400	-	Puerarin	Chicken embryo	Liga et al. (2024)
35	Parkinson’s Disease	NIR-assisted MgO-based polydopamine nanoparticles	160 (hydrodynamic diameter)	−15.0	Puerarin, pDNA	Mouse	Gao et al. (2022)
36	-	Gold nanoparticles	19–20	−4.47	Puerarin	-	Zehra et al. (2021)
37	Alcoholic hepatitis	Mesoporous silicon nanoparticles	267.7 ± 98.9	−3.546 ± 0.7774	Puerarin	Mouse	Zhang et al. (2022b)
38	Ophthalmic diseases	Poly (2-hydroxyethyl methacrylate-co-methacrylated hyaluronan-β-cyclodextrin) hydrogel	-	−20.1–35.5	Puerarin/Levofloxacin	-	Deng et al. (2024)
39	Ophthalmic diseases	Poly (methacrylated-PVA-co-mono-methacrylated-beta-cyclodextrin) hydrogels	-	-	Puerarin	-	Xu et al. (2010)
40	Ophthalmic diseases	Poloxamer analogues/carbopol-based *in situ* gelling	-	-	Puerarin	Rabbit	Qi et al. (2007)
41	Ischaemic stroke	Chitosan hydrogel	132.92 ± 8.44	−20.23 ± 3.26	Puerarin/Ac2-26	Rat	Xu et al. (2025)
42	Hind limb ischaemia	GelMA hydrogel	-	-	Puerarin	Mouse	Pan et al. (2023)
43	Wound healing	Polyethylene glycol diacrylate hybrid hydrogel	108–204	-	Puerarin	Rat	Zhang et al. (2019b)
44	Wound healing	Cell-penetrating peptide–poly (lactic-co-glycolic acid)	134.57 ± 1.42	2.14 ± 0.78	Puerarin	Mouse	Lin et al. (2024)
45	Wound healing	Polyethylene glycol diacrylate hydrogel	193–233	-	Puerarin/Ferulic acid/Polydopamine	Rat	Ou et al. (2021)
46	Myocardial infarction	Injectable chitosan hydrogel	∼170	-	Puerarin/Silica nanoparticles	Rat	Feng et al. (2023)
47	Wound healing	Carboxymethyl chitosan/sodium alginate oxide-based hydrogel	253.7	−23.2 ± 1.2	Puerarin	Mouse	Zhai et al. (2025)
48	Periodontitis	Probiotic hydrogel	∼50	−5.51 ± 0.58	Puerarin	Rat	Wang et al. (2025d)
49	-	Chitosan hydrogel	59.67	-	Puerarin	-	Xie et al. (2024)
50	Antibacterial	Thiolated chitosan/puerarin composite hydrogel	-	-	Berberine chloride Hydrate/Puerarin	-	Yuan et al. (2024)
51	Antibacterial	Chitosan hydrogel	4.62	-	Berberine Chloride Hydrate/Puerarin	-	Yuan et al. (2022)
52	Wound healing	Silk fibroin methacrylated and modified collagen type III ROS‐responsive hydrogels	217.7 ± 17.1	-	Puerarin	Rat	Yang et al. (2024c)
53	Tendon injury	Chitosan hydrogel	∼74	-	Puerarin	Rat	Wan et al. (2024)
54	Ulcerative colitis	Chitosan and β-sodium glycerophosphate thermosensitive hydrogel	164.58 ± 1.75	−32.0 ± 0.6	Puerarin/Zein/Bletilla striata polysaccharide	Mouse	Zhao et al. (2024)
55	Myocardial infarction	Neutrophil membrane	210.43 ± 2.25	−19.03 ± 0.87	Puerarin	Mouse	Wang et al. (2025e)
56	Ischaemic stroke	Cubic liquid crystal	274.70 ± 16.20	−25.30 ± 2.34	Puerarin	Rat	Chen et al. (2024c)
57	Acute myocardial ischaemia	RGD-modified and PEGylated solid lipid nanoparticles	110.5	−26.2	Puerarin	Rat	Dong et al. (2017)
58	Myocardial infarction	Solid lipid nanoparticles	112.6 ± 3.1	−30.3 ± 2.7	Puerarin–pro-drug–tanshinone	Rat	Guo et al. (2019)
59	-	Nanostructured lipid carriers	159 ± 1.1	−28.3	Puerarin	-	Ye et al. (2021)
60	-	Solid lipid nanoparticles	160	−35.43	Puerarin	Rat	Luo et al. (2011)
61	-	Lipid nanocapsules	181.0	23.8	Puerarin	-	Wang et al. (2017b)
62	-	β-CD/PAA/PEG polymer micelles	39.2–322.0	−19.6 to −29.7	Puerarin	-	Hu et al. (2016)
63	Antiapoptotic	PEG–PE micelles	16.0	−24.1	Puerarin	Rat	Li et al. (2018)
64	Diabetic nephropathy	Hybrid micelles based on natural polysaccharides	173.1	−24.43	Puerarin	Mouse	Guo et al. (2023)
65	-	Chitosan (2-hydroxyl-3-butoxyl)–propylcarboxymethyl-chitosan micelles	400	-	Puerarin	-	Weiping et al. (2006)
66	Ophthalmic diseases	Poly (amido amine) dendrimer complex	-	-	Puerarin	Rabbit	Wang et al. (2011)
67	Ophthalmic diseases	Poly (amidoamine) dendrimers	-	-	Puerarin	Rabbit	Yao et al. (2010a)
68	Ophthalmic diseases	Poly (amidoamine) dendrimers	-	-	Puerarin	Rabbit	Yao et al. (2010b)
69	-	Poly (amidoamine) dendrimers	-	-	Puerarin	Rat	Gu et al. (2013)
70	-	Nanocrystals	229.7	−46	Puerarin	Dog	Yi et al. (2015)
71	-	Nanocrystals	∼20	∼0	Puerarin	Rat	Cheng et al. (2020)
72	Parkinson’s disease	Nanocrystals	83.05 ± 1.96	−22.41 ± 1.19	Puerarin	Zebrafish	Xiong et al. (2019)
73	-	Nanocrystals	525.8	-	Puerarin	Rat	Tu et al. (2013)
74	Myocardial ischaemia	Nanocrystals	20–40	-	Puerarin	Rat	Tu et al. (2020)
75	-	Hydroxypropyl-β-cyclodextrin	264.4 ± 9.8	-	Puerarin	-	Zhang et al. (2021c)
76	-	Solid self-microemulsifying drug delivery system	20.37	−23.5	Puerarin	Rat	Cheng et al. (2016b)
77	-	Microemulsion	40.2 ± 5.9	-	Puerarin	Rat	Wu et al. (2011)
78	-	N-trimethyl chitosan-modified microemulsion	20.69–25.07	-	Puerarin	Rat	Liao et al. (2015)
79	-	Microemulsions	13.50–24.04	-	Puerarin	Rat	Tang et al. (2013)
80	-	Microemulsions	40.2 ± 5.9	-	Puerarin	Mouse	Wu et al. (2009)

### 4.1 Nanoparticles

Nanoparticles have been extensively explored as drug-delivery systems and possess several advantages, such as their small size, ability to penetrate cell membranes and blood vessel walls, high drug-loading efficiency, and controllable physicochemical properties (Maghsoudi et al., 2020; Kashani et al., 2024). By optimizing the particle structure and properties, controlled drug release may be achieved, potentially improving therapeutic performance and reducing adverse effects (Men et al., 2020; Moradi Kashkooli et al., 2020). Moreover, the use of biocompatible and biodegradable materials may improve the biocompatibility profile of the carrier (Severino et al., 2019). Drug encapsulation may protect payload molecules from damage caused by external factors such as enzymatic degradation and changes in pH, improving drug stability (Rao et al., 2015). Surface modification may reduce immune recognition and prolong systemic circulation, thereby increasing drug exposure time *in vivo* (Bamberger et al., 2017).

#### 4.1.1 Polymer nanoparticles

Polymer nanoparticles are composed of different hydrophilic and hydrophobic moieties, typically with particle sizes ranging from 10 to 1000 nm. They can simultaneously encapsulate both hydrophilic and hydrophobic drugs (Thakur et al., 2023). Due to its limited aqueous solubility, puerarin can be effectively encapsulated within polymer nanoparticles (Markwalter et al., 2019). Commonly used polymer nanoparticles include PLGA and PEG (Saraswat et al., 2016; Xiao et al., 2018). Such carriers feature a high specific surface area, adjustable pore structures, tunable molecular weights, and flexible preparation processes (Thakur et al., 2023). In addition, nanoparticle size, surface charge, and drug-release behavior can be regulated (Huang et al., 2020; Yan et al., 2020), potentially enabling controlled puerarin release and influencing its tissue distribution profile, which may improve renal exposure and therapeutic efficacy. Moreover, the surface of the polymer nanoparticles can be modified (Oroojalian et al., 2017), which may facilitate the development of cell-specific renal targeting strategies for puerarin.

#### 4.1.2 Inorganic nanoparticles

Inorganic nanoparticles (such as silver, gold, platinum, zinc oxide, iron oxide, and cerium oxide) typically have particle sizes ranging from 10 to 100 nm. They possess unique physicochemical properties and controllable surface functionalization capabilities and have been widely investigated for drug-delivery applications (Jiao et al., 2018). For example, iron oxide nanoparticles exhibit magnetic responsiveness (Feng et al., 2006; Nowak-Jary and Machnicka, 2022). Puerarin could potentially be incorporated into such systems, and external magnetic guidance may provide a strategy for site-selective drug delivery. However, its applicability to kidney-targeted puerarin delivery remains to be experimentally validated.

Compared with inorganic nanoparticles, polymer nanoparticles may offer advantages such as biodegradability and favorable biocompatibility profiles (Eltaib, 2025). Nevertheless, inorganic nanoparticles possess unique properties, including magnetic responsiveness and surface functionalization capabilities, which may provide additional opportunities for future drug-delivery applications.

### 4.2 Hydrogels

Hydrogels are three-dimensional network-structured polymer systems formed by monomers containing hydrophilic functional groups through simple reactions. They have a diameter range of 20–10,000 nm and physical properties similar to those of human soft tissues (Merino et al., 2015). Their water-absorbing capacity stems from the hydrophilic functional groups on the polymer backbone, while their water insolubility is achieved through the cross-linking of network chains (Ai et al., 2023). Compared with conventional hydrogels, nanogels may offer higher drug-loading capacity, improved release characteristics, and enhanced permeability in certain applications, and a larger specific surface area available for modification (Delgado-Pujol et al., 2025).

#### 4.2.1 Supramolecular hydrogels

Supramolecular hydrogels are formed through noncovalent intermolecular interactions and have a broad drug encapsulation capacity (Cheng X. et al., 2016). Puerarin can be encapsulated within supramolecular hydrogels, and their pH responsiveness and mucoadhesive properties may provide opportunities for site-selective drug release (Liu W. et al., 2023). For instance, hydroxypropyl-β-cyclodextrin-g-poly (acrylic acid)/gelatine semi-interpenetrating network hydrogels possess pH-dependent swelling and mucoadhesive properties. After puerarin loading, these hydrogels have been reported to exhibit pH-responsive swelling and controlled drug-release behavior, which may be advantageous for site-selective delivery. Moreover, their mucoadhesive properties may prolong local retention and potentially influence therapeutic effect (Naeem et al., 2023).

#### 4.2.2 Stimuli-responsive hydrogels

Stimuli-responsive hydrogels have high water content, high sensitivity, and controllable structures and physicochemical properties (Rezanejade Bardajee et al., 2025). They can rapidly respond to external environmental stimuli such as temperature, light, magnetic field, pH, ionic strength, and ATP. Different stimulus-responsive hydrogels may be considered according to the pathological characteristics of kidney diseases and the microenvironment of potential target sites (Fang et al., 2023). For example, in view of the differences in the pH of different segments of the renal tubule, pH-responsive hydrogels may represent a potential option, which may undergo physicochemical changes and modulate drug-release behavior under specific pH conditions (Ma et al., 2024). In cases where precise control of the drug release time is needed, light-responsive hydrogels may provide an additional strategy. External light stimulation may enable temporal control of drug release, improving treatment accuracy.

### 4.3 Liposomes

Liposomes are hollow spherical structures formed by a phospholipid bilayer enclosing an aqueous phase region. They are commonly used nanocarrier systems, typically with particle sizes ranging from 50 to 200 nm. Liposomes possess both hydrophilic and hydrophobic domains and can effectively encapsulate water-soluble and lipid-soluble drugs (Nsairat et al., 2022). Liposomes themselves usually do not have renal-targeting ability (Nsairat et al., 2022), and active-targeting ligands may be conjugated to their surface to achieve the renal-targeted delivery of puerarin. For example, for targeted modification of endothelial cells and MCs, 3,5-dipentyloxybenzamidine hydrochloride (TRX-20) can be added to the surface of liposomes; this imparts a positive charge to the liposomes, and TRX-20 has an affinity for chondroitin sulfate proteoglycans, thus forming a liposomal drug carrier with targeting functions for endothelial cells and MCs. Such a strategy may facilitate increased local drug exposure in damaged tissues. TRX-20-targeted liposomes with a particle size of approximately 100 nm can increase the deposition of the drug in the glomeruli and spleen of healthy rats (Morimoto et al., 2007). In addition, the delivery of anti-inflammatory drugs using TRX-20-targeted liposomes has achieved certain results in related studies. For instance, the delivery of triptolide can improve the abnormal levels of proteinuria and serum ALB in a rat model of membranous nephropathy (Yuan et al., 2017). These findings support the feasibility of TRX-20-modified liposomal systems as a potential platform for puerarin delivery.

### 4.4 Micelles

When the critical micelle concentration is reached, micelles are thermodynamically stable aggregates self-assembled by surfactants in aqueous solutions, usually with particle sizes in the range of 10–100 nm (Figueiras et al., 2022). They have a hydrophobic core and a hydrophilic outer layer and can physically encapsulate hydrophobic drugs through hydrophobic interactions and hydrogen bonding (Guerrero-Hernández et al., 2022). As a hydrophobic drug, puerarin can be effectively loaded into the core of micelles in this way, which may improve its aqueous solubility, stability, and bioavailability (Shang et al., 2024). For example, chrysophanol-loaded micelles have a particle size of 29.64 ± 0.71 nm. In addition to accumulating in the kidneys, they also accumulate in the brain and liver. *In vitro* and *in vivo* experiments revealed that the cumulative release rate of chrysophanol in a chrysophanol-loaded micellar system is significantly greater than that in a free drug system and that chrysophanol has a good protective effect on podocytes (Li et al., 2018). These studies provide a basis for the rational design of puerarin-loaded micellar systems.

### 4.5 Dendrimers

Dendrimers are highly branched, monodisperse macromolecules formed by the repeated branching of low-molecular-weight polymers. Their particle size typically ranges from 1 to 100 nm. Dendrimers consist of a core, a polymer backbone, and dendritic unit side chains, with an internal cavity structure and abundant reactive functional groups such as amino and hydroxyl groups on the surface. Puerarin may be incorporated into dendrimers through physical encapsulation within the internal cavity or through covalent interactions between surface functional groups and drug molecules, potentially improving drug-loading efficiency and stability (Li et al., 2018).

Dendrimers generally exhibit favorable aqueous solubility and may demonstrate acceptable biocompatibility following appropriate surface modification (Matsuura et al., 2018). Through surface engineering, their physicochemical properties may be tailored to support renal-targeting applications. In addition, by using the reactive functional groups on the surface of dendrimers to connect renal-targeting ligands, such as ligands specific to the surface receptors of MCs and TECs, such ligands may facilitate cell-specific delivery of puerarin. For instance, according to research on polyamidoamine (PAMAM) dendrimers, S-nitrosylated L-serine is modified on the surface of PAMAM dendrimers to construct a renal-targeted nitric oxide (NO) donor. In a mouse model of renal I/R injury, this carrier effectively inhibited the increase in plasma creatinine levels, the expression of kidney injury markers, and histological changes (Matsuura et al., 2018). Dendrimer-based systems represent a platform for future puerarin delivery strategies. Puerarin-loaded dendrimers incorporating renal-targeting ligands may represent a potential strategy for future kidney-targeted delivery.

### 4.6 Other nanomaterials

NCs increase the dissolution rate and bioavailability of puerarin by reducing its particle size to the nanoscale (20–150 nm). This property is attributed to the high specific surface area effect and enhanced surface free energy of the nanocrystals, which increase the likelihood that the drug molecules will dissolve and be rapidly absorbed (Macedo et al., 2024). Puerarin nanocrystals are prepared by high-pressure homogenization using polyvidone K30 as a stabilizer, and nanocrystals with a particle size of 20–50 nm can be obtained (Tu et al., 2020). This particle size range may be compatible with certain passive renal-targeting strategies. For example, curcumin nanocrystals inhibited ferroptosis and alleviated kidney injury by restoring GPX4 expression in a diabetic nephropathy model (Xue M. et al., 2025), suggesting potential utility for renal drug-delivery applications. These studies suggest that nanocrystal technology may represent an effective strategy for improving puerarin delivery. The puerarin microemulsion system is a thermodynamically stable nanodispersion system (usually with a particle size of 20–200 nm) that is spontaneously formed by water, oil, surfactants, and cosurfactants (Cheng G. et al., 2016). The surface of the microemulsion can be modified, such as by attaching hyaluronic acid targeting the CD44 receptor on the surface of renal MCs or lactoferrin targeting the megalin receptor of the proximal tubule, which may facilitate receptor-mediated cellular uptake and influence local drug release (Hu et al., 2018). Self-emulsifying systems (SEDDSs) are prenanocarriers that spontaneously form microemulsions with a particle size of <100 nm in the gastrointestinal environment. SEDDSs consist of an oil phase (such as medium-chain triglycerides), an emulsifier (such as polyoxyethylene castor oil), and a coemulsifier (such as PEG 400) (Tu et al., 2020). After encountering water, they spontaneously forms nanoemulsion droplets driven by low interfacial energy; SEDDSs may improve oral absorption and alter the biodistribution of puerarin through lymphatic transport pathways.

Collectively, dendrimers, nanocrystals, microemulsions, and self-emulsifying systems provide diverse material platforms for the future development of kidney-targeted puerarin delivery. However, most available evidence originates from non-renal disease models or studies involving therapeutic agents other than puerarin. Therefore, their applicability to kidney-targeted puerarin delivery remains largely prospective and requires further validation in relevant renal disease models.

## 5 Conclusion and future Perspectives

Puerarin has significant pharmacological potential in the treatment of renal diseases through its antioxidant, anti-inflammatory and antifibrotic effects. However, the poor aqueous solubility, rapid systemic clearance, and unfavorable pharmacokinetic properties of puerarin substantially limit its renal exposure and therapeutic efficacy. Therefore, the rational design of nanocarrier systems for puerarin should focus on improving drug solubility, prolonging circulation time, enhancing renal biodistribution, and achieving controlled release.

Despite encouraging preclinical findings, several limitations should be acknowledged. Current evidence supporting the renoprotective effects of puerarin is derived from *in vitro* studies and animal models, while clinical evidence remains limited. Moreover, commonly used experimental models may not fully recapitulate the complexity and heterogeneity of human kidney diseases. The doses employed in preclinical studies are often substantially higher than those achievable in clinical settings, raising concerns regarding dose translation and pharmacokinetic relevance. Therefore, the therapeutic efficacy and long-term safety of puerarin in human renal diseases remain to be fully established.

The physiological ability of the kidney to excrete drugs poses a great challenge for drug delivery to renal tissues (Zhou et al., 2014). It is challenging for experts in the pharmaceutical field to design ideal delivery platforms to ensure that the drugs reach the kidneys efficiently and work at the target site. Some nanocarrier materials are potentially toxic, such as the surface cations of dendritic polymers, which may lead to cytotoxicity and haemolytic toxicity (Roszkowski and Durczynska, 2025), and inorganic nanoparticles are poorly biodegradable and may accumulate in the body, triggering a long-term toxic response (Jakic et al., 2024). In addition, nonspecific protein adsorption and unintended immune activation may alter nanoparticle biodistribution, accelerate clearance, and increase the risk of adverse biological responses, further complicating the safety assessment of nanomedicines (Mahmoudi et al., 2023). Although toxicity can be reduced to a certain extent by surface modification and the selection of biodegradable materials, the extent of bioaccumulation of nanocarriers after long-term repeated administration, the effect on metabolic degradation pathways in the liver and kidney, and the mechanism of organ transport of nanomedicines with different physicochemical properties, remain incompletely understood and continue to present important challenges for the biosafety assessment of nanomedicines.

Future studies should focus on the rational design of puerarin-specific nanocarriers to facilitate the translation of kidney-targeted nanomedicines. Direct comparisons between free puerarin and nanocarrier-based formulations are needed to determine whether nanoformulations can achieve meaningful improvements in pharmacokinetic behavior, renal biodistribution, tissue retention, and therapeutic efficacy. Advanced imaging approaches and biomarker-guided strategies may further facilitate the evaluation of renal targeting efficiency and intrarenal distribution. In addition, comprehensive assessment of long-term biosafety, including chronic toxicity and immunogenicity, remains essential before clinical application. The therapeutic potential of puerarin-loaded nanocarriers should also be validated across multiple renal disease models, including AKI, DN, and CKD, to address disease-specific differences. Furthermore, successful translation will require overcoming challenges related to large-scale manufacturing, batch-to-batch reproducibility, formulation stability, quality control, and regulatory approval. Establishing standardized production protocols and evaluation criteria will therefore be critical for ensuring consistent product quality and facilitating clinical development.

Overall, nanotechnology provides promising opportunities to optimize the delivery of puerarin. Future formulation strategies should be tailored to the physicochemical and pharmacokinetic characteristics of puerarin to maximize renal accumulation while minimizing systemic exposure and off-target effects. Therefore, further interdisciplinary efforts integrating pharmacology, materials science, nanotechnology, and nephrology are required to establish safe, effective, and clinically translatable kidney-targeted puerarin delivery systems. Accordingly, the kidney-targeting strategies summarized in this review should be regarded as prospective approaches rather than established delivery systems for puerarin, and their feasibility requires experimental validation in future studies.

## Glossary

ABCG2: ATP-binding cassette subfamily G member 2
AGEs: Advanced glycation end products
AKI: Acute kidney injury
AKT: RAC-alpha serine/threonine-protein kinase
AMPK: 5′-AMP-activated protein kinase
ANT: Adenine nucleotide translocator
AREs: Antioxidant response elements
ATF4: Cyclic AMP-dependent transcription factor ATF-4
Atg5: Autophagy protein 5
Bax: Apoptosis regulator BAX
Bcl-2: Apoptosis regulator Bcl-2
BCL6: B-cell lymphoma 6 protein homologue
CaO: Calcium oxalate
CHOP: DNA damage-inducible transcript 3 protein
CKD: Chronic kidney disease
COL: Collagen
CREB: Cyclic AMP-responsive element-binding protein
CRF: Chronic renal failure
CTGF: CCN family member 2
Cyt c: Cytochrome c
DJ-1: Parkinson disease protein 7
DN: Diabetic nephropathy
ECM: Extracellular matrix
eIF2α: Eukaryotic translation initiation factor 2A
eNOS: Endothelial nitric oxide synthase
ERS: Endoplasmic reticulum stress
ESRD: End-stage renal disease
FA: Fatty acid
FN: Fibronectin
FOXO1: Forkhead box protein O1
GFB: Glomerular
GFB: Glomerular filtration barrier
GLUT9: Glucose transporter 9
Gnai1: Guanine nucleotide-binding protein G(i) subunit alpha-1
GRP78: Endoplasmic reticulum chaperone BiP
GSDMD: Gasdermin D
GSH-Px: Glutathione peroxidase
HO-1: Haem oxygenase-1
I/R: Ischaemia‒reperfusion
IL: Interleukin
JAK2: Tyrosine protein kinase JAK2
JNK: c-Jun N-terminal kinase
KEAP1: Kelch-like ECH-associated protein 1
LC3: Light chain 3
lncRNA: Long noncoding RNA
MCP-1: Monocyte chemoattractant protein-1
MCs: Mesangial Cells
MDA: Malondialdehyde
miRNA: MicroRNA
mPTP: Mitochondrial permeability transition pore
mTOR: Mechanistic target of rapamycin
MTX: Methotrexate
MyD88: Myeloid differentiation primary response protein MyD88
NC: Nanocrystal
NF-κB: Nuclear factor-κB
NLRP3: LRR and PYD domains-containing protein 3
NO: Nitric Oxide
NOX4: NADPH oxidase 4
Nrf2: Nuclear factor erythroid 2-related factor 2
OAT1: Solute Carrier Family 22 Member 6
OAT3: Solute Carrier Family 22 Member 8
PAMAM: Polyamidoamine
PARP: Poly(ADP-ribose) polymerase
PEG: Polyethylene glycol
PERK: Protein kinase R-like ER kinase
PGC-1α: Peroxisome proliferator-activated receptor γ coactivator 1-α
PGE2: Prostaglandin E2
PI3K: Phosphatidylinositol 3-kinase
PKCβ: Protein kinase C beta type
PLGA: Poly(lactic-co-glycolic acid)
PNP: Polymeric nanoparticles
PP2A: Protein phosphatase 2A
PPARα: Peroxisome Proliferator Activated Receptor α
RAASis: Renin–angiotensin–aldosterone system inhibitors
RAB7: Ras-related protein Rab-7a
RAGE: Advanced glycation end products
ROS: Reactive oxygen species
SEDDSs: Self-emulsifying drug delivery systems
SIRT1: Sirtuin-1
Smad: SMAD family member
SOD: Superoxide dismutase
STAT3: Signal transducer and activator of transcription 3
STZ: Streptozotocin
TECs: Renal tubular epithelial cells
TGF-β: Transforming growth factor-β
TLR4: Toll-like receptor 4
TNF-α: Tumour necrosis factor-α
TRX - 20: 3,5-Dipentyloxybenzamidine hydrochloride
UUO: Unilateral ureteral obstruction
VCAM-1: Vascular Cell Adhesion Molecule-1
α-SMA: α-smooth muscle actin
